# Erucic Acid, Derived by *Lactobacillus Crispatus*, Induces Ferroptosis in Cervical Cancer Organoids Through the PPAR‐δ Signaling Pathway

**DOI:** 10.1002/advs.202512599

**Published:** 2025-10-13

**Authors:** Qianwei Zhen, Yuexuan Xu, Ying Xu, Xiao Liu, Yuting Zhang, Dan Ye, Sai Han, Shili Liu, Youzhong Zhang

**Affiliations:** ^1^ Department of Obstetrics and Gynecology Qilu Hospital of Shandong University 107 Wenhua Xi Road Jinan Shandong Province 250012 China; ^2^ Department of Microbiology School of Basic Medical Sciences Cheeloo College of Medicine Shandong University 44 Wenhua Xi Road Jinan Shandong Province 250012 China; ^3^ Shandong Key Laboratory of Reproductive Health and Birth Defects Prevention and Control 44 Wenhua Xi Road Jinan Shandong 250012 China

**Keywords:** cervical cancer organoids, erucic acid, ferroptosis, lactobacillus crispatus, PPAR‐δ, ROS

## Abstract

The microbiome present throughout the human body serves a variety of functions. In this study, 16S rRNA sequencing is employed to uncover differences in the abundance of *Lactobacillus* within the vaginal microbiota between individuals with cervical cancer and those with healthy cervixes. The research further identifies that the metabolite of *Lactobacillus crispatus* can induce ferroptosis in cervical cancer cells. This conclusion is reached through targeted bacterial culture, patient‐derived organoids (PDO) and single‐cell RNA sequencing. Erucic acid, identified as a primary metabolite via untargeted metabolomics, acts as a ligand for PPARδ receptor. It has the capacity to activate PPARδ pathway and subsequently trigger downstream fatty acid oxidation (FAO). Excessive enhancement of FAO can generate large amounts of H_2_O_2_ and O_2_‐, known as ROS. Utilizing PDO, cell lines and cervical cancer xenograft (CDX) models, the study demonstrate both in vitro and in vivo that the metabolite of *L. crispatus*, erucic acid, can modulate the proliferation, migration and invasion of cervical cancer by activating the PPAR‐δ pathway. This activation leads to fatty acid oxidation, release ROS, and ultimately induces ferroptosis. Therefore, *L. crispatus* and erucic acid show potential as novel adjuvant therapeutic agents in the treatment of cervical cancer.

## Graphical abstract

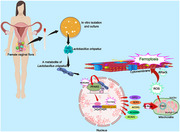

## 1 Introduction

The incidence and mortality rates of cervical cancer (CC) rank the fourth among all female cancers globally. As vaccination against cervical cancer^[^ ^1^ ^]^ and the implementation of effective screening methods become more widespread,^[^ ^2^ ^]^ a reduction in CC incidence is anticipated in the near future. However, these preventative measures are not compulsory, and the vaccines do not cover all subtypes of human papillomavirus (HPV). In 2018, there were 569 847 new cases of CC diagnosed worldwide, and 311 365 deaths.^[^ ^3^ ^]^ By 2020, these figures had risen to 604 000 new cases and 342 000 deaths. Consequently, the primary challenge now is to either cure CC or prevent the progression of cervical intraepithelial neoplasia (CIN) to CC. Therefore, the development of enhanced treatment strategies is of paramount importance.^[^ ^4^ ^]^

A multitude of studies have established a link between the urogenital microbiome and the progression of HPV infections and gynecological malignancies, especially CC.^[^ ^5^, ^6^, ^7^ ^]^ The majority of these investigations indicate that the vaginal microbiota could act as biomarkers for CC and CIN, offering innovative approaches for the detection, prevention, and diagnosis of cervical abnormalities.^[^ ^8^ ^]^ Various microorganisms, such as *Neisseria, Lactobacillus, Prevotella, Propionibacterium, Lachnoanaerobaculum, Streptococcus, Veillonella, Capnocytophaga, Campylobacter*, and *Fusobacterium*, have been identified as potential enhancers or inhibitors for CC.^[^ ^9^ ^]^ However, there is a scarcity of in‐depth research on how the vaginal microbiota influences cervical health, which is crucial for considering the vaginal microbiota as a therapeutic strategy for CC or CIN.^[^ ^10^ ^]^ Most researchers concur that a *Lactobacillus*‐rich vaginal microbiota can preserve a healthy vaginal milieu, thus counteracting HPV infections and the progression of CIN.^[^ ^7^, ^11^, ^12^ ^]^ *Lactobacillus*, as a prospective biotherapeutic agent for cervical cancer treatment, has been shown to significantly impede cancer cell migration and invasion, and to promote apoptosis, making its potential use in cervical cancer treatment promising, although the precise mechanisms are yet to be elucidated.

On the contrary, a recent study by Colbert et al. has suggested that *Lactobacillus iners* is correlated with poorer patient survival rates, the induction of chemoresistance and radioresistance in cervical cancer cells, and the triggering of metabolic reprogramming or shifts in multiple tumor metabolic pathways.^[^ ^13^ ^]^ This indicates that not all *Lactobacillus* species are beneficial for female health. In addition, Colbert et al also found differences of *Lactobacillus iners* in the vaginal environment between healthy cervix women and cervical cancer patients, and there seemed to be some mutations of *Lactobacillus iners*. This reminds us that bacteria, as microorganisms, are extremely prone to genetic mutations and thereby changes in biological behaviors. In clinical practice, there are some vaginal suppositories that directly use live *Lactobacillus* as medicines. Are these bacteria safe and reliable? Will these bacteria undergo genetic mutations and harm the health of patients? In light of these findings, it is imperative to conduct detailed studies on the specific strains of *Lactobacillus* to comprehend their precise impacts and the mechanisms at play. Such research is also essential for the broad clinical application of vaginal probiotic therapies.^[^ ^11^ ^]^

To delve deeper into the mechanisms by which the vaginal microbiota affects cervical lesions, establishing a more sophisticated cervical cancer model is essential. Despite this, cervical cancer cell lines continue to be the predominant model in laboratory settings. The unique anatomy of the cervix poses challenges in developing animal models with cervical tumors, which has impeded more profound research into cervical cancer. Organoids, which are 3D tumor models derived from the proliferation and self‐organization of cells directly isolated from patient tumor tissue, offer a promising alternative. The advent of cancer organoids has facilitated a more accurate representation of the natural heterogeneity of cancer cells found within actual tumors, thus preserving the tumor's pathophysiology in vitro. This includes maintaining the genetic and phenotypic traits that are specific to certain types of tumors.^[^ ^14^ ^]^ Organoids, based on their genetic blueprint, autonomously develop into 3D structures that more closely mimic the actual growth and progression of tumors.^[^ ^15^ ^]^ This technology provides a valuable tool for studying the intricate interactions between the vaginal microbiota and cervical lesions, potentially leading to breakthroughs in our understanding of cervical cancer development and progression. By leveraging the advantages of organoid models, researchers can better explore the complex biological processes at play, paving the way for more effective diagnostic and therapeutic strategies.

In this research, we successfully established patient‐derived organoid (PDO) models of cervical cancer based on methodologies outlined in previous studies.^[^ ^16^, ^17^, ^18^, ^19^, ^20^ ^]^ The PDO models encompassed both high‐risk HPV‐positive (designated as PDO‐HPV16‐a and PDO‐HPV16‐b) and intermediate‐risk HPV‐positive (PDO‐HPV82) organoids. Subsequently, we conducted a comprehensive analysis of the metabolites produced by *Lactobacillus crispatus* (indicative of the healthiest vaginal state). We treated the PDOs with bacterial culture supernatants and focused on erucic acid, a metabolite that exhibited the most significant yield variance. Our findings indicated that these treatments could inhibit the growth of PDOs, and the mechanism by which erucic acid arrests cell growth was subsequently further investigated. Metabolites are more easily prepared than bacteria, as they involve fewer genetic mutations and pose less biological toxicity to the host. Therefore, drugs derived from metabolites are likely to be safer and more reliable. This study represents a significant step forward in utilizing organoid technology to model and study the complex interactions between the vaginal microbiome and cervical cancer progression, offering new insights into potential preventative and therapeutic interventions.

## 2 Results

### 2.1 Differential Presence of *Lactobacillus* in Vaginal Microbiota Between Cervical Cancer Patients and Healthy Cervical Women, as well as the Isolation of *L. crispatus* (*LC 001*)

**Figure 1:**
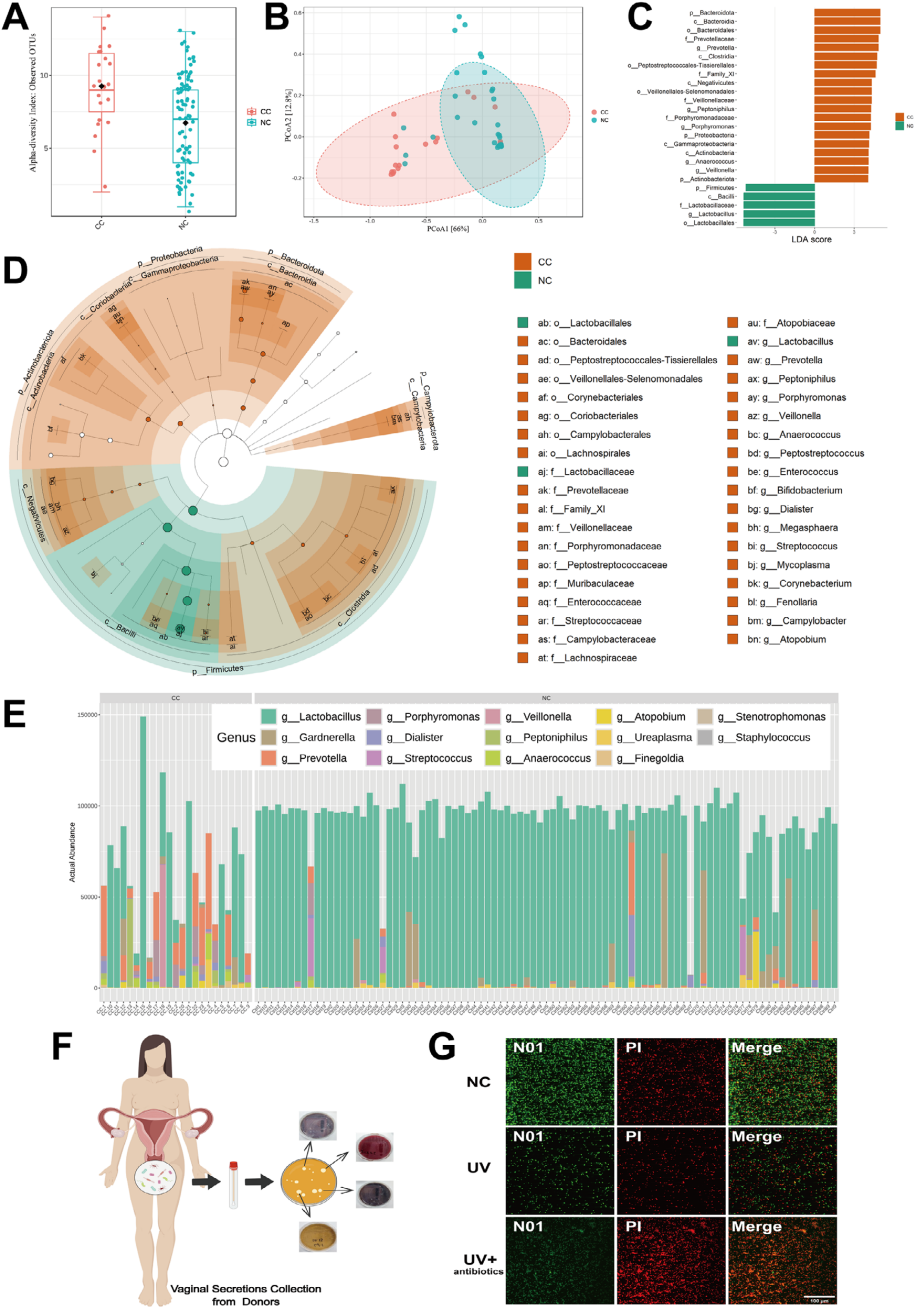
Comparison of vaginal microbiota differences between cervical cancer women and healthy cervical women through 16S rRNA sequencing. A) α‐ diversity of 23 cervical cancer patients and 89 women with healthy cervices. Unpaired *t*‐test (two‐tailed). *p <* 0.001. B) β‐diversity of 23 cervical cancer patients and 89 women with healthy cervices. PCoA1 *p <* 0.0001; PCoA2 p = 0.1422. C,D) Lefse analysis revealed differences in the dominant bacterial genera in the vaginal microbiota of women with cervical cancer compared to those with healthy cervices. E) The bar chart suggests that the levels of *Lactobacillus* in the vaginal microbiota of cervical cancer patients are significantly lower than those in women with healthy cervices. F) The procedure for isolating the vaginal microbiota: Uniformly spread the vaginal secretions of healthy cervical women onto a common bacterial culture medium. After several days, locate the delicate, smooth and white colonies (*Lactobacillus*) and spread them onto MRS agar. (G) UV: Expose the *L. crispatus* to UV light continuously for 1 h; UV+ antibiotic: First, expose the *L. crispatus* to UV light for 1 h, then add 1% antibiotics and incubate overnight. N01/PI fluorescent staining: N01 can stain live bacteria with green fluorescence and PI can stain dead bacteria with red fluorescence.

To explore the influence of the vaginal microbiome on cervical cancer development, this research gathered 16S rRNA sequencing data from vaginal secretions of 23 cervical cancer patients and 89 women with healthy cervices (Tables S1 and S2, Supporting Information). According to clinical diagnostic criteria, the normal range of vaginal secretion pH is 3.8–4.5. In this study, 61 cases (68.54%) and 7 cases (30.43%) in the NC group and CC group, respectively, had pH values within this normal range. The pH values of the remaining cases, 28 cases (31.46%) and 16 cases (69.57%), were all above 4.5. The results of the 16S rRNA sequencing indicated a substantial disparity in the vaginal microbiota composition between the two groups. Notable variations were observed in both α‐ and β‐diversity (as depicted in **Figure** **1**A,B). Further analysis using Lefse revealed a predominance of the genus *Lactobacillus* and its superior taxon (*p__Firmicutes; c__Bacilli; o__Lactobacillales; f__Lactobacillaceae; g__Lactobacillus*) in the healthy controls, which were less prevalent in cervical cancer patients. Conversely, the genera *Prevotella* (*p__Bacteroidetes; c__Bacteroidia; o__Bacteroidales; f__Prevotellaceae; g__Prevotella*)*, Peptoniphilus* (*p__Firmicutes; c__Clostridia; o__Clostridiales; f__Clostridiaceae; g__Peptoniphilus*)*, Porphyromonas* (*p__Bacteroidetes; c__Bacteroidia; o__Bacteroidales; f__Porphyromonadaceae; g__Porphyromonas*)*, Veillonella* (*p__Firmicutes; c__Clostridia; o__Clostridiales; f__Veillonellaceae; g__Veillonella*) and *Anaerococcus* (*p__Firmicutes; c__Clostridia; o__Clostridiales; f__Clostridiaceae; g__Anaerococcus*), as well as their superior taxons, were found to be more abundant in the latter group (as illustrated in Figure 1C,D). This observation was corroborated by a bar chart highlighting a significant reduction in *Lactobacillus* levels among individuals with CC (Figure 1E). These results indicate that the vaginal microbiota of healthy cervices is predominantly composed of *Lactobacillus*, with very little proportion occupied by other genera. However, the composition of the vaginal microbiota in cervical cancer patients is complex and chaotic, with a significant number of bacterial genera dominating the microbiota that are not present in healthy cervices. We speculate that this phenomenon may be due to chronic vaginal bleeding in cervical cancer patients disrupting the balance of the vaginal microbiota, although the underlying mechanisms may not be that simple. It is well‐known that the vaginal environment of healthy women is weakly acidic, primarily due to the presence of large numbers of *Lactobacillus*. Additionally, *Lactobacillus* can resist the colonization of other pathogenic bacteria, thus preventing the occurrence of vaginal diseases. Given that most cervical cancers in clinical settings are exophytic, protruding beyond the normal cervical epithelium, this morphological feature places the cancer directly within the envelopment of the vaginal microbiota. Therefore, we believe that *Lactobacillus* must have certain effects on the occurrence and development of cervical cancer. The vaginal microbiota can be categorized into five Community State Types (CSTs). Among these, CST‐I, characterized by the predominance of *Lactobacillus crispatus* in the vagina, is indicative of the healthiest vaginal state and is commonly observed in most healthy women. Numerous studies have shown that when vaginal health is compromised, the dominant microbiota shifts from *L. crispatus* to other species such as *L. iners*, and even to opportunistic pathogens. In addition, the probability of isolating *L. crispatus* is the highest when we perform vaginal *Lactobacillus* cultures. Therefore, we selected *L. crispatus* over other bacteria for this study. To delve deeper into the potential link between *Lactobacillus* and cervical cancer, the study successfully isolated *L. crispatus* (*LC 001*) from the vaginal secretions of healthy controls in vitro (Figure 1F), which was identified through DNA sequencing (refer to Figure S1, Supporting Information). Subsequent active fluorescence staining of the in vitro cultured *L. crispatus* (as shown in Figure 1G) indicated that the bacteria were in an excellent state.

### 2.2 Cervical Cancer Organoids are Very Similar to the Primary Tumors in both Morphology and Biomarkers

**Figure 2:**
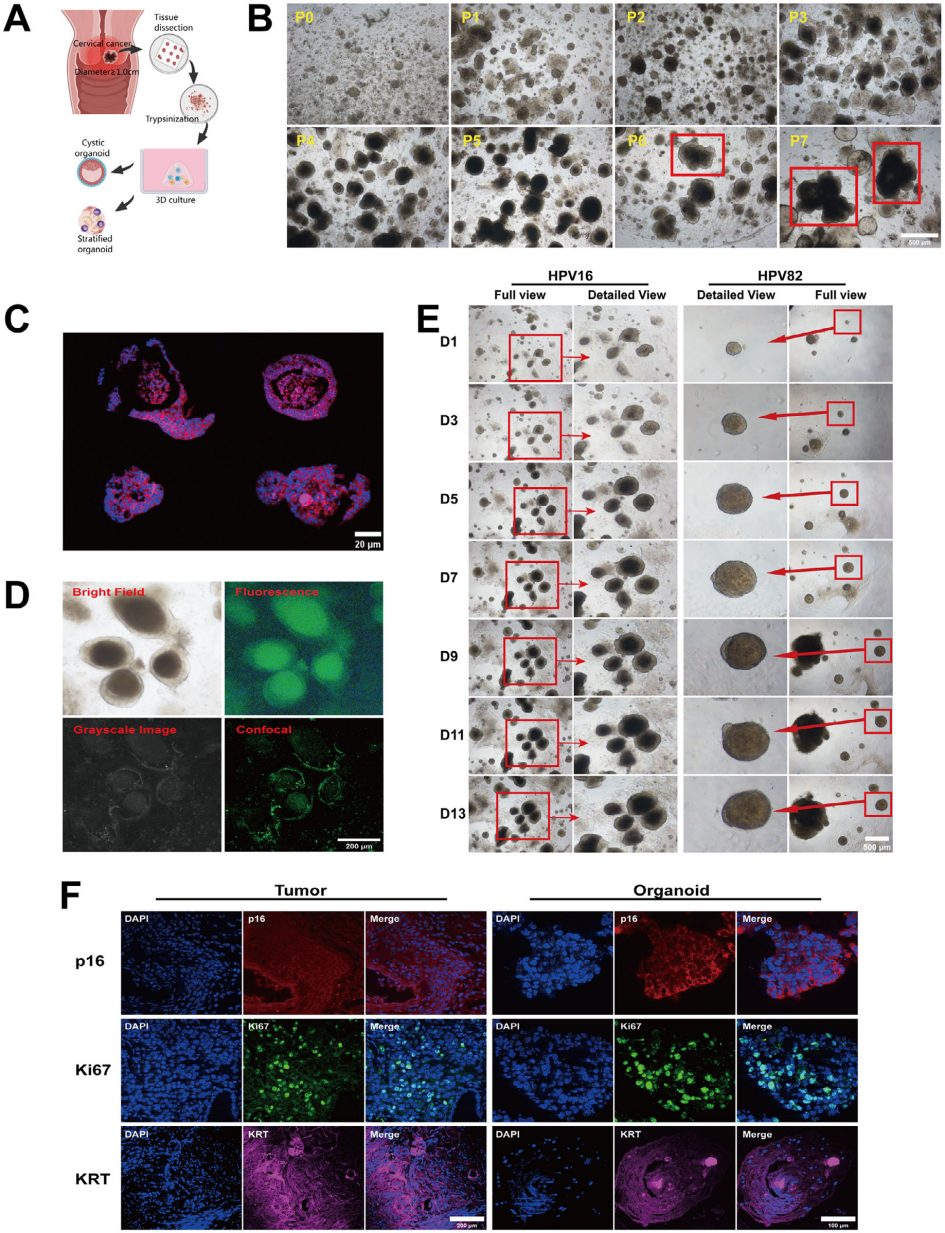
Establishment of cervical cancer organoid models. A) Method for collecting cervical cancer tissue to create organoids. B) Bright view of cervical cancer organoids from P0 to P7 under the microscope. Inside red rectangular box: cauliflower‐like structures of cervical cancer organoids. Scale bar, 500 µm. C) Morphology of cervical cancer organoids. Blue fluorescence: hoechst 33 342; red fluorescence: phalloidin‐AF555. Scale bar, 20 µm. D) Organoids after lentivirus infection exhibit green fluorescence. Scale bar, 200 µm. E) Bright view of PDO‐HPV16 and PDO‐HPV82 from D1 to D13 under the microscope. Inside red rectangular box: gradually growing organoids. Scale bar, 500 µm. F) Immunohistofluorescence staining results of proteins relating to tumors. The organoids and their originating tumors exhibited positive expression for the three proteins p16, ki67, and KRT. Blue fluorescence: DAPI. Scale bar for tumor, 200 µm. Scale bar for organoid, 100 µm.

To elucidate the potential influence of *L. crispatus* on cervical cancer progression, we successfully established cervical cancer organoids for both high‐risk and medium‐risk HPV‐positive cases. The cervical cancer sources we selected must meet the requirement (Table S2, Supporting Information) that the tumors’ diameters were at least 1 cm to avoid affecting pathological diagnosis due to our sampling procedure (**Figure** **2**A). These organoids were subculture for multiple generations, and still maintaining high viability as demonstrated in Figure 2B. They possess unique spatial structures, resulting in potential gaps between the inner core and outer wall of certain organoids, while others appear to be completely solid (Figure 2C). We consider them to be mature organoids when the diameter of the organoids can grow to at least 200 µm and up to a maximum of 500 µm through ≈13 days of culture (Figure 2E). Our cultured cervical cancer organoids were categorized into parenchymal and vesicular types (Figure 2B), just like those cultured by Hans et al.^[^ ^17^ ^]^ Notably, with each subculture, the organoids' morphology increasingly resembled that of cauliflower‐like structures, which is characteristic of clinical cervical cancer tumors (Inside the red rectangular box of Figure 2B). This observation underscores the reliability of our organoid model in mimicking the physiological state observed in clinical settings. Furthermore, standard techniques were employed to enable the lentivirus to infect these mature organoids, and subsequent microscopic examination revealed robust fluorescence, indicating that these organoids are amenable to serve as valuable models for further investigations (Figure 2D). In the present research, three distinct cervical cancer organoid models were utilized: PDO‐HPV82, which was positive for HPV82 (medium‐risk HPV); PDO‐HPV16‐a and PDO‐HPV16‐b, both of which were positive for HPV16 (high‐risk HPV). Cervical cancer organoids, like their originating cervical cancer tissues, tested positive for p16, Ki67 and KRT, demonstrating that the organoids share similar biological behavior characteristics with the source cervical cancer tissues (Figure 2F).

### 2.3 The Cell‐Free Supernatant Rather than Bacterial Pellet of *L. crispatus* Inhibited the Growth of Cervical Cancer Both In Vitro and In Vivo

**Figure 3:**
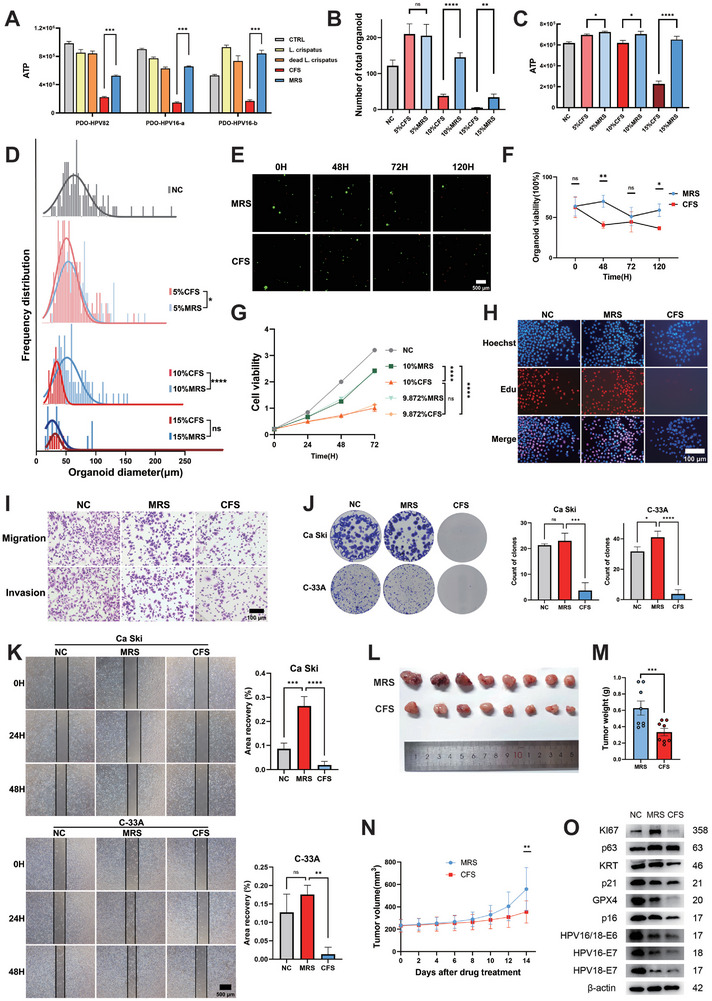
CFS inhibited the growth of cervical cancer both in vitro and in vivo. A) Divided the organoids into 5 groups after 300 g centrifution to obtain the sediment, and then each group received the following treatments: CTRL: add PBS whose volume was the same to other groups to one group of organoids; *L. crispatus*: added the lived *L. crispatus* which was resuspended in PBS with the same volume as the PBS of the CTRL group to the organoids to ensure the MOI = 50; dead *L. crispatus*: added the dead *L. crispatus* which had been exposed to UV light for 1 h and 1% antibiotics incubated overnight to ensure the MOI = 50; CFS: Added *L. crispatus* to the liquid MRS medium and cultivated them in an anaerobic environment at 37 °C, 190 rpm for at least 72 h until the bacterial density reached ≈1 × 10^9^ cells mL^−1^. Centrifuged the culture at 4000 rpm for 40 min at 20 °C and then sterile‐filtered the culture with 20 µm cell strainers. MRS: added pure liquid MRS medium to one group of organoids. All groups’ volume was same. Statistical analyses: 2way ANOVA, ***p < 0.001. B) The number of organoids treated with MRS and CFS from 5% to 15%. Statistical analyses: paired t tests, ns *p >* 0.05, **p < 0.01, ****p < 0.0001. C) The ATP levels of organoids treated with MRS and CFS from 5% to 15%. Statistical analyses: paired t tests, *p < 0.05, ****p < 0.0001. D) Particle‐size analysis of organoids treated with MRS and CFS from 5% to 15%. Statistical analyses: paired t tests, ns *p >* 0.05, *p < 0.05, ****p < 0.0001. E) AO/PI Mixed Dye analysis of organoids treated with MRS and CFS from 0 to 120 h. Green fluorescence: lived organoids. Red fluorescence: dead organoids. Scale bar, 500 µm. F) The viability of organoids treated with MRS and CFS from 0 to 120 h tested by MONWEI SmartCell 800. Statistical analyses:2way ANOVA, ns *p >* 0.05, *p < 0.05, **p < 0.01. G) Cell Counting Kit‐8 assays of Ca Ski treated by NC, 10%MRS, 10%CFS, 9.872%MRS and 9.872%CFS, respectively. Statistical analyses: unpaired t tests, ns *p >* 0.05, ****p < 0.0001. H) Edu assays of Ca Ski cell lines treated with 10%PBS, 10%MRS and 10%CFS. Blue fluorescence: Hoechst (nuclei of all the cells). Red fluorescence: 5‐Ethynyl‐2′‐deoxyuridine (proliferating cells). Scale bars, 100 µm. I) Transwell assays of Ca Ski cell lines treated with 10%PBS, 10%MRS and 10%CFS. Scale bars, 100 µm. J) Cell clonogenic assays of Ca Ski and C‐33A cervical cancer cell lines treated with 10%PBS, 10%MRS and 10%CFS. Statistical analyses: paired t tests, ***p < 0.001, ****p < 0.0001. K) Scratch assays of Ca Ski and C‐33A cervical cancer cell lines treated with 10%PBS, 10%MRS and 10%CFS. Statistical analyses: paired t tests, ns *p >* 0.05, **p < 0.01, ***p < 0.001, ****p < 0.0001. L) Pictures of the tumors treated with MRS and CFS in cell derived xenograft (CDX) models. M) Bar chart of tumor weights after MRS and CFS treatment. Statistical analyses: paired t tests, ***p < 0.001. N) Bar chart of tumor volume after MRS and CFS treatment. Statistical analyses: paired t tests, **p < 0.01. O) Western Blot results relating to proliferation and HPV of Ca Ski treated with 10%PBS, 10%MRS and 10%CFS.

In our investigation into the influence of *L. crispatus* on cervical cancer, we co‐cultured the three cervical cancer organoids, PDO‐HPV82 (HPV82‐positive), PDO‐HPV16‐a (HPV16‐positive), and PDO‐HPV16‐b (HPV16‐positive), with *L. crispatus*. To clarify the mechanisms through which *Lactobacillus crispatus* influences cervical cancer, we subjected cervical cancer organoids to cell‐free supernatant (CFS is the supernatant of bacterial culture medium in which *L. crispatus* has been cultured, containing metabolites produced by *L. crispatus*) and pellet of *L. crispatus* (only bacteria, no culture medium). The latter included live bacteria and dead bacteria (dead bacteria were inactivated by UV irradiation and antibiotic treatment). Control groups included PBS control (CTRL) and blank culture medium (MRS, bacterial culture medium without any microorganisms has been cultured). The ATP assay for organoid viability was evaluated 24 h after treatment, and the results showed that ATP levels in organoids treated with CFS were significantly lower than those in other groups (**Figure** **3**A), indicating that it was not the *L. crispatus* bacteria but the metabolites of *L. crispatus* that suppressed cervical cancer. Consequently, we co‐cultured newly subcultured cervical cancer organoids PDO‐HPV16‐a with CFS and MRS at concentration gradients of 5%, 10%, and 15% and monitored their growth under a microscope for 21 consecutive days (Figure S2, Supporting Information). The data indicated that significant differences between CFS and MRS treatments in the number of PDO‐HPV16‐a organoids were observed at the 10% and 15% concentration (Figure 3B). ATP analysis of organoids revealed differences at all three concentration gradients, with the most pronounced effects at 15% (Figure 3C). However, organoid particle size showed significant differences at both 5% and 10% concentrations (Figure 3D). Based on these findings, we selected a 10% CFS concentration for subsequent treatments and performed dead/alive fluorescence staining (Figure 3E) and viability analysis (Figure 3F) on PDO‐HPV16‐a organoids treated at different time points. The number of dead organoids (red fluorescence) after CFS treatment was significantly higher than in the MRS treatment group, with the most substantial difference observed at 48 h. Therefore, all subsequent studies used 48 h as the co‐culture time, including the cell lines.

To observe the effect of CFS on different cervical cell lines, Ca Ski, C‐33A, Hela and H8 cell lines were treated with 10% CFS, and the cell morphology was observed under a microscope. It was found that the morphological changes of Ca Ski cells were most obvious, and the cells treated with CFS showed obvious shrinkage and distortion. It seemed to have more shrunken and dead cells of C‐33A cells in the CFS group. However, there was no obvious morphological change in Hela and H8 cell lines (Figure S4A, Supporting Information). Therefore, subsequent cell experiments were mainly conducted on Ca Ski cells, supplemented by C‐33A cells.

In order to exclude the influence of pH value on the experimental results, we tested the pH value of reagents (Table S3, Supporting Information). In addition, because MRS does not contain bacterial metabolites, the pH value of MRS is higher than that of CFS. After measurement, the pH value of 10%MRS is equivalent to that of 9.872% CFS. Therefore, we treated the Ca Ski cells with 10%MRS and 9.872%CFS, and used 10%CFS and 9.872%MRS as controls to test cell viability (Figure 3G). The results showed that there was no significant statistical difference between 9.872%CFS and 10%CFS in the viability of cervical cancer cell lines, while there was a significant statistical difference between 9.872%CFS and 10%MRS. This showed that the difference in the effect of 10%MRS and 10%CFS on cervical cancer was attributed to the metabolite rather than variation in pH values. Therefore, it is scientifically rigorous to conduct the experiment using 10%CFS as the experimental group and 10%MRS as the control group.

Afterward, we used 5‐Ethynyl‐2′‐deoxyuridine (Edu) (Figure 3H) and transwell experiments (Figure 3I) to evaluate the effect of CFS on Ca Ski (HPV‐16 positive) and found that CFS could significantly weaken the ability of Ca Ski in proliferation, migration and invasion. Furthermore, we incubated Ca Ski and C‐33A (HPV negative) cell lines with CFS for 48 h and then performed the cell clonogenic assays (Figure 3J) and scratch assays (Figure 3K). The final results showed that both HPV positive cancer cells (Ca Ski) and HPV negative cancer cells (C‐33A) were suppressed by CFS. Their proliferation and migration abilities were weakened by CFS. We further validated the inhibitory effects of CFS on cervical cancer in cell derived xenograft (CDX) models. 16 female BALB/cAnN‐Foxn1nu/nu/Rj mice, aged 4–5 weeks, were subcutaneously injected with an equal amount of Ca Ski cells to establish subcutaneous tumors. The CDX models were treated with MRS and CFS once the tumor growth was confirmed to be well‐established (volume ≥100mm^3^). Our findings revealed that by the 14th day following the initial treatment, the tumors in the CFS group were significantly smaller compared to the MRS group, both in weight and volume (Figure 3L, M,N). We then detected the levels of proliferation‐related and HPV‐related proteins in Ca Ski by Western Blot and found that most of them showed a trend of decreased vitality (Figure 3O). This suggests that CFS not only have cytotoxic effects on cervical cancer in vitro but also exhibit significant killing effects in vivo.

### 2.4 Single‐Cell Sequencing Analysis Enriched Cellular Processes Related to Ferroptosis in Organoids Treated with CFS of *L. crispatus*

**Figure 4:**
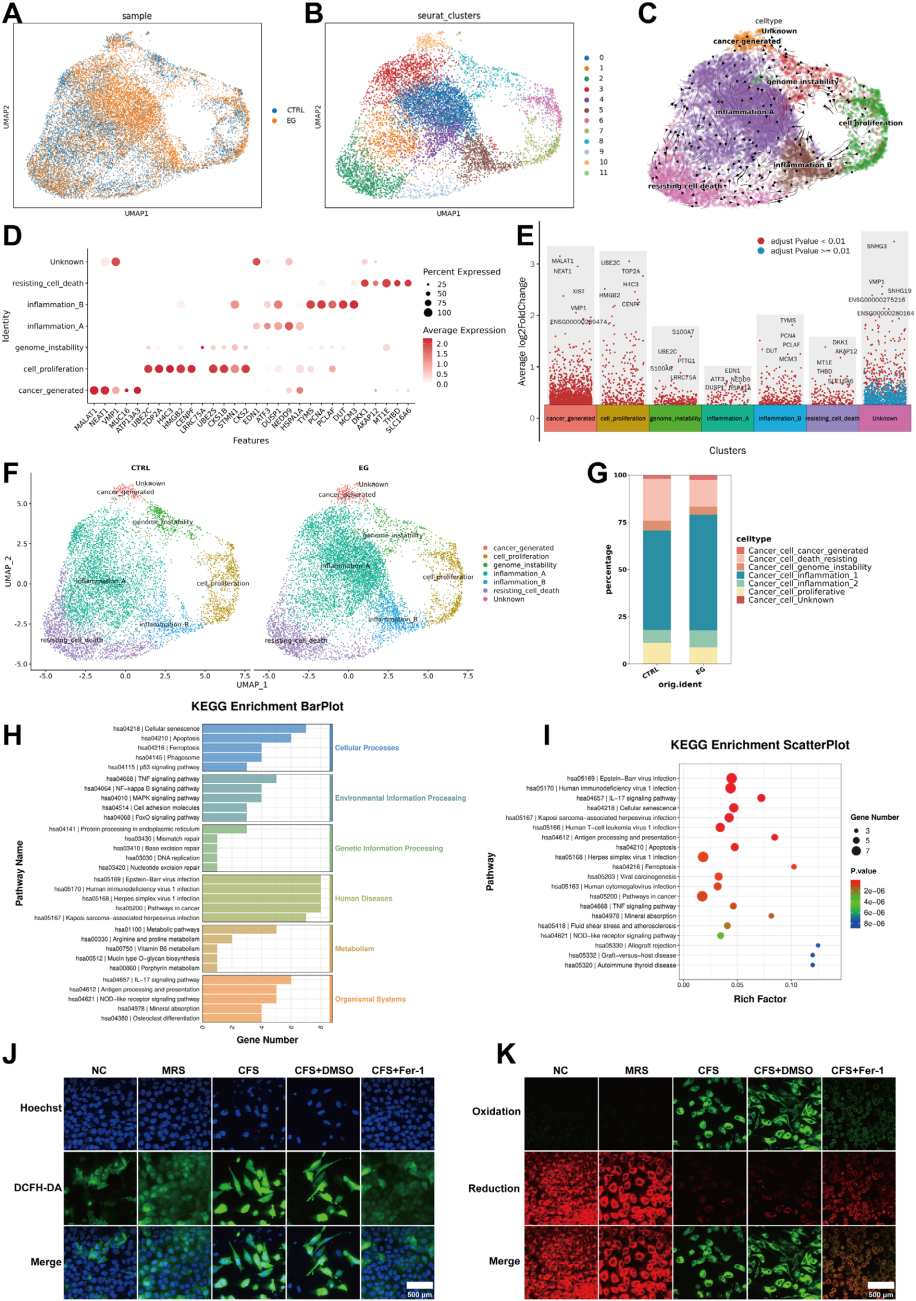
Single‐cell sequencing analysis of organoids. A) UMAP of EG and CTRL. B) Seurat clusters of all the single cells of organoids. C) Velocity‐streamlines analysis of every cell cluster. D,E) Some certain molecules were significant different in the expression levels within different clusters. F) UMAP of new cell clusters basing on cell types. G) The percentage of every cluster between EG and CTRL. H) KEGG enrichment barplot of pathway name of the “cell death resisting” cluster between EG and CTRL. I) KEGG enrichment scatterplot of the “cell death resisting” cluster between EG and CTRL. J) ROS assays of Ca Ski cell lines treated with 10%PBS, 10%MRS (MRS is the bacterial culture medium without any microorganisms has been cultured), 10%CFS (CFS, is the supernatant of bacterial culture medium in which *L. crispatus* has been cultured, containing metabolites produced by *L. crispatus*), 10%CFS+DMSO (DMSO is the solvent of Fer‐1) and 10%CFS+Fer‐1. Blue fluorescence: Hoechst (nuclei of all the cells). Green fluorescence: ROS. Scale bars, 500 µm. K) C11‐Bodipy assays of Ca Ski cell lines treated with 10%PBS, 10%MRS, 10%CFS, 10%CFS+DMSO and 10%CFS+Fer‐1. Green fluorescence: oxidation. Red fluorescence: reduction. Scale bars, 500 µm.

To elucidate the underlying mechanisms by which *L. crispatus* exerts its effects on cervical cancer, we performed single‐cell sequencing on organoids treated with MRS and CFS. The cervical cancer organoids were dissociated into individual living cells, which were then subjected to individual analysis. Initially, the organoid samples were categorized using the UMAP algorithm, and the results revealed distinct clustering patterns between the experimental group (EG) and control groups (CTRL) (**Figure** **4**A). Based on these patterns, the samples were broadly sub‐classified into 12 clusters (Figure 4B). Notably, there were significant differences in the expression levels of certain molecules among cells within different clusters (Figure 4D). The molecules with the most pronounced expression variations included MALAT1, NEAT1, XIST, UBE2C, TOP2A, H4C3, HMGB2, and SNHG3, among others (Figure 4E). Based on the UMAP clustering results, we used the functional scores of the 50 HallMark pathways in ssGSEA to evaluate the feature genes in each cluster to determine the tumor cell types. Therefore, by combining the ssGSEA heatmap, we performed clustering of the characteristic scores for different clusters to judge the functional types of tumor cells and further stratify the organoid samples. Two types of inflammatory tumor cells (inflammation A & inflammation B) were identified because during clustering, it was found that there were certain differences between cluster 5 and the other four clusters expressing inflammatory characteristics, so they were labeled as two different types of inflammatory tumor cells (Figure 4F). Comparative analysis of the cell populations between the treatment and control groups indicated a marked increase in the proportion of inflammation‐associated cells in the experimental group, while the proportion of cells involved in cell proliferation and resistance to cell death decreased, particularly those that are able to resist cell death. This observation aligns with the organoid functional assays conducted in the earlier phase of this study (Figure 4G). Velocity‐streamlines analysis also revealed that cells exhibiting characteristics of resistance to cell death and cell proliferation were diverging. The direction of the arrows in the diagram indicated that there was a flow relationship of mutual transformation between the various clusters of cells (Figure 4C). What's more, the pseudotime analysis of seurat clusters (Figure S3A,C, Supporting Information) and new cell clusters basing on cell types (Figure S3B,D, Supporting Information) suggested that, in both MRS group and CFS group, the cells of cluster 10, cluster 8, “cancer generated” and “genome instability” were at the end of the monocle‐tree diagram, indicating that these cells may be fully mature or specialized in the developmental path. These cells may have reached their final developmental state and no longer undergo further differentiation. While the other cell populations were almost evenly distributed on the branches of the monocle‐tree diagram, indicating that they underwent further cell differentiation after CFS treatment and may play a role in certain physiological processes. To delve deeper into the mechanisms behind these changes, we conducted a separate analysis of the cell death resistance subgroups within both the treatment and control groups. KEGG pathway enrichment analysis identified numerous cellular processes associated with cell death, with ferroptosis associated factors being the most significantly enriched (Figure 4H,I). To verify that CFS can induce ferroptosis in cervical cancer organoids, we conducted ROS assays (which detect reactive oxygen species levels, a key indicator of ferroptosis) and C11‐bodipy assays (a quantitative method for assessing ferroptosis) on Ca Ski cell lines treated with 10% PBS, 10% MRS, 10% CFS, 10% CFS + DMSO, and 10% CFS + Fer‐1 (Ferrostatin‐1, a ferroptosis inhibitor), respectively. The results indicated that the levels of ROS (Figure 4J) and ferroptosis (Figure 4K) in cervical cancer cells treated with 10% CFS and 10% CFS + DMSO were significantly elevated, and this increase could be effectively reversed by the ferroptosis inhibitor Fer‐1. Thus, it is evident that CFS of *L. crispatus* can indeed induce ferroptosis in cervical cancer cells.

### 2.5 Erucic Acid, the Metabolite of *L. crispatus*, Might Be Involved in Inducing Ferroptosis in Cervical Cancer

**Figure 5:**
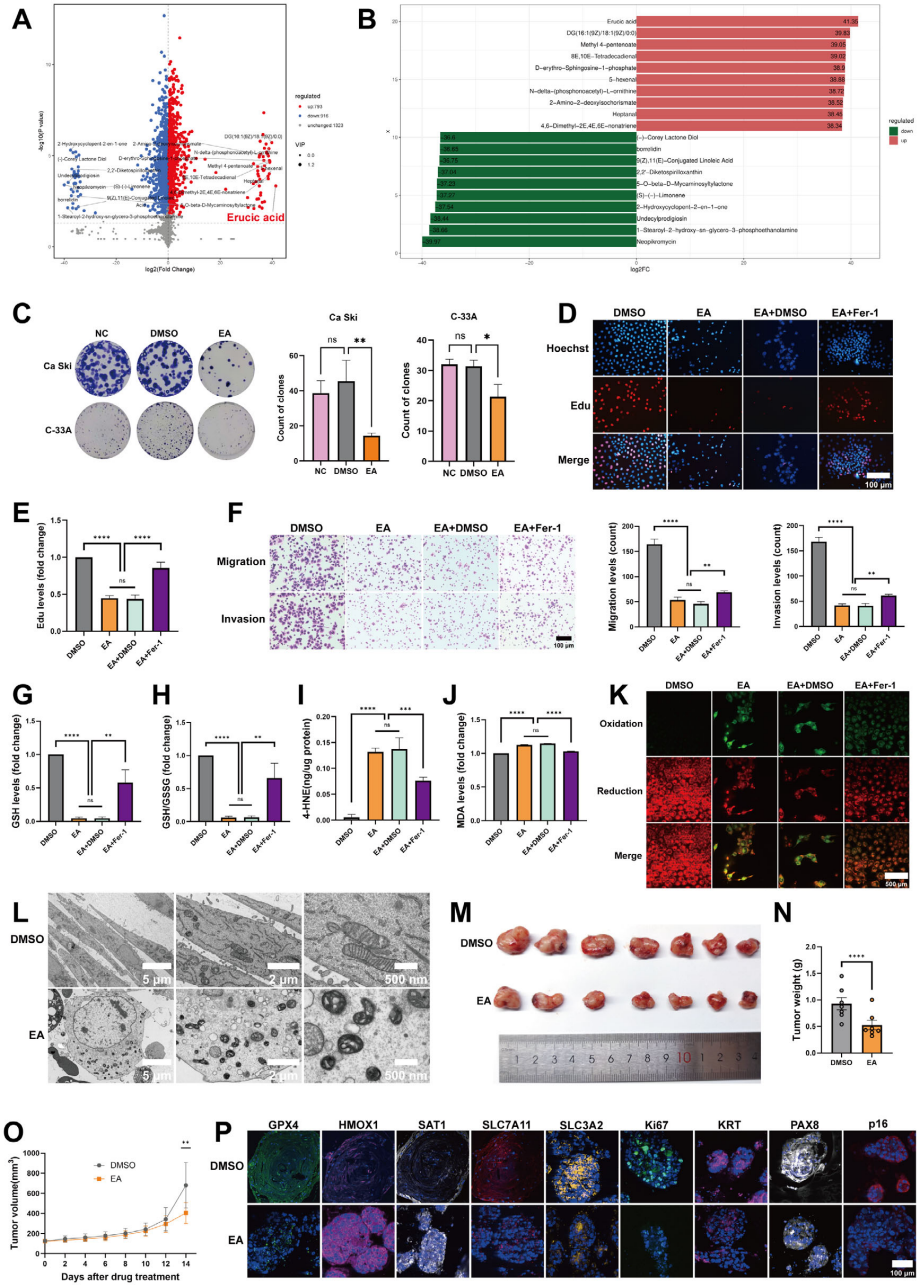
Erucic acid inhibits cervical cancer viability by inducing ferroptosis. A) Volcano plot of all the metabolites in untargeted metabolomics analysis from CFS. B) The barplot of the top 20 metabolites with the most significant increases and decreases. C) Cell clonogenic assays of Ca Ski and C‐33A cervical cancer cell lines treated with EA and DMSO. Statistical analyses: paired t tests, ns *p >* 0.05, *p < 0.05, **p < 0.01. D) Edu assays of Ca Ski cell lines treated with DMSO, EA, EA+DMSO and EA+Fer‐1. Blue fluorescence: Hoechst (nuclei of all the cells). Red fluorescence: 5‐Ethynyl‐2′‐deoxyuridine (proliferating cells). Scale bars, 100 µm. E) Bar chart of the results of Edu assays. Statistical analyses: paired t tests, ns *p >* 0.05, ****p < 0.0001. F) Transwell assays of Ca Ski cell lines treated with DMSO, EA, EA+DMSO and EA+Fer‐1. Scale bars, 100 µm. Statistical analyses of bar chart: paired t tests, ns *p >* 0.05, **p < 0.01, ****p < 0.0001. G) Bar chart of the GSH levels (fold change). Statistical analyses: paired t tests, ns *p >* 0.05, **p < 0.01, ****p < 0.0001. H) Bar chart of the ratio of GSH and GSSG (fold change). Statistical analyses: paired t tests, ns *p >* 0.05, **p < 0.01, ****p < 0.0001. I) Bar chart of the levels of 4‐HNE. Statistical analyses: paired t tests, ns *p >* 0.05, ***p < 0.001, ****p < 0.0001. J) Bar chart of the levels of MDA. Statistical analyses: paired t tests, ns *p >* 0.05, ****p < 0.0001. K) C11‐Bodipy assays of Ca Ski cell lines treated with DMSO, EA, EA+DMSO and EA+Fer‐1. Green fluorescence: oxidation. Red fluorescence: reduction. Scale bars, 500 µm. L) The morphology of mitochondria of cervical cancer cells treated with EA under the transmission electron microscopy. Scale bars, 5 µm, 2 µm, 500 nm. M) Pictures of the tumors treated with DMSO and EA in cell derived xenograft (CDX) models. N) Bar chart of tumor weights after DMSO and EA treatment. Statistical analyses: paired t tests, ****p < 0.0001. O) Bar chart of tumor volume after DMSO and EA treatment. Statistical analyses: paired t tests, **p < 0.01. P) Immunohistofluorescence staining results relating to ferroptosis and proliferation of cervical organoids treated with DMSO and EA. Scale bars, 100 µm.

To identify the possible substances responsible for inducing ferroptosis in cervical cancer organoids, we performed an untargeted metabolomics analysis of the CFS from *L. crispatus*. The results showed that the levels of 1323 metabolites did not change, while the numbers of metabolites with increased and decreased levels were 793 and 916, respectively. Among the top 20 metabolites that showed the most significant increase and decrease in content (**Figure** **5**A), erucic acid stood out as the metabolite with the highest increase in CFS production by *L. crispatus* (Figure 5B).

Afterward, we treated Ca Ski cells with erucic acid of different concentration gradients and observed the cell morphology under a microscope. We found that Ca Ski had obvious shrinkage and death at a concentration of 250 µm (Figure S4B, Supporting Information). In addition, the CCK‐8 assays of Ca Ski and C‐33A cells also confirmed the above results (Figure S4C,S4D, Supporting Information). Therefore, the concentration of 250 µm was used in all subsequent experiments.

To explore the effect of EA (erucic acid) on cervical cancer viability, we conducted a series of experiments. Cell clonogenic assays showed that erucic acid had an inhibitory effect on both HPV‐positive (Ca Ski) and HPV‐negative (C‐33A) cervical cancer (Figure 5C). The Edu experiment verified that EA can inhibit the proliferation of cervical cancer, and this inhibitory effect can be partially rescued by Fer‐1 (Ferrostatin‐1, a ferroptosis inhibitor) (Figure 5D,E). Transwell experiments showed that EA could also reduce the migration and invasion ability of cervical cancer, which could also be partially rescued by Fer‐1 (Figure 5F). The aforementioned findings indicate that EA might suppress the proliferative, migratory, and invasive capacities of cervical cancer cells via the induction of ferroptosis. A similar effect was previously observed when cervical cancer cells were treated with CFS derived from *L. crispatus*, suggesting that EA could be one of the active components of CFS. To verify the occurrence of ferroptosis, we detected various ferroptosis‐related products, including GSH (reduced glutathione), GSSG (oxidized glutathione disulfide), 4‐HNE (4‐Hydroxynonenal) and MDA (Malondialdehyde). The GSH level and GSH/GSSG ratio in cervical cancer cells treated with EA were significantly decreased, and this reduction trend could be partially reversed by Ferrostatin‐1, which aligns with glutathione depletion in ferroptosis (Figure 5G,H). As products of ferroptosis, the levels of 4‐HNE and MDA were significantly elevated in EA‐treated cervical cancer cells, and the increasing trend could also be mitigated by Ferrostatin‐1 (Figure 5I,J). In addition, the C11‐Bodipy assay also indicated a significant rise in ferroptosis levels in cells treated with EA, followed by a decrease upon Ferrostatin‐1 treatment (Figure 5K). Under the transmission electron microscopy, the mitochondria of EA‐treated cervical cancer cells exhibited shrinkage, increased mitochondrial membrane density, and degeneration or disappearance of mitochondrial cristae, consistent with the microscopic characteristics of ferroptosis (Figure 5L). Subsequently, we administered the same dose of EA and solvent DMSO into the subcutaneous tumors of cell‐derived xenograft (CDX) models, following the same experimental protocol as in Figure 3L. After a 14‐day observation period, the volume and weight of the tumor in the EA group were significantly lower than those in the solvent DMSO group (Figure 5M, N,O). The above results demonstrate that EA can inhibit the activity of cervical cancer both in vivo and in vitro. We further examined ferroptosis‐related markers and cancer proliferation‐related markers in cervical organoids, revealing that EA‐treated organoids exhibited a trend of enhanced ferroptosis and reduced vitality (Figure 5P).

### 2.6 Erucic Acid Induces Ferroptosis via PPAR‐δ Pathway

Erucic acid is known to act as a ligand for the PPARδ receptor and can activate the PPAR‐δ pathway, which is associated with downstream lipid metabolism‐related signals, as indicated by the KEGG database. These signals include lipid synthesis, cholesterol metabolism, fatty acid transport and fatty acid oxidation. Ferroptosis is a non‐apoptotic form of programmed cell death characterized by the uncontrolled accumulation of lipid peroxidation, a chain reaction triggered by ROS (reactive oxygen species) attacking polyunsaturated fatty acids in cytomembranes. The main endogenous sources of ROS are the ETC (electron transport chain) and FAO (fatty acid oxidation). Excessive FAO produces a large amount of NADH (Reduced Nicotinamide Adenine Dinucleotide), which drives the ETC and aggravates electron leakage. A large number of leaked electrons are directly transferred to oxygen to produce ROS.

**Figure 6:**
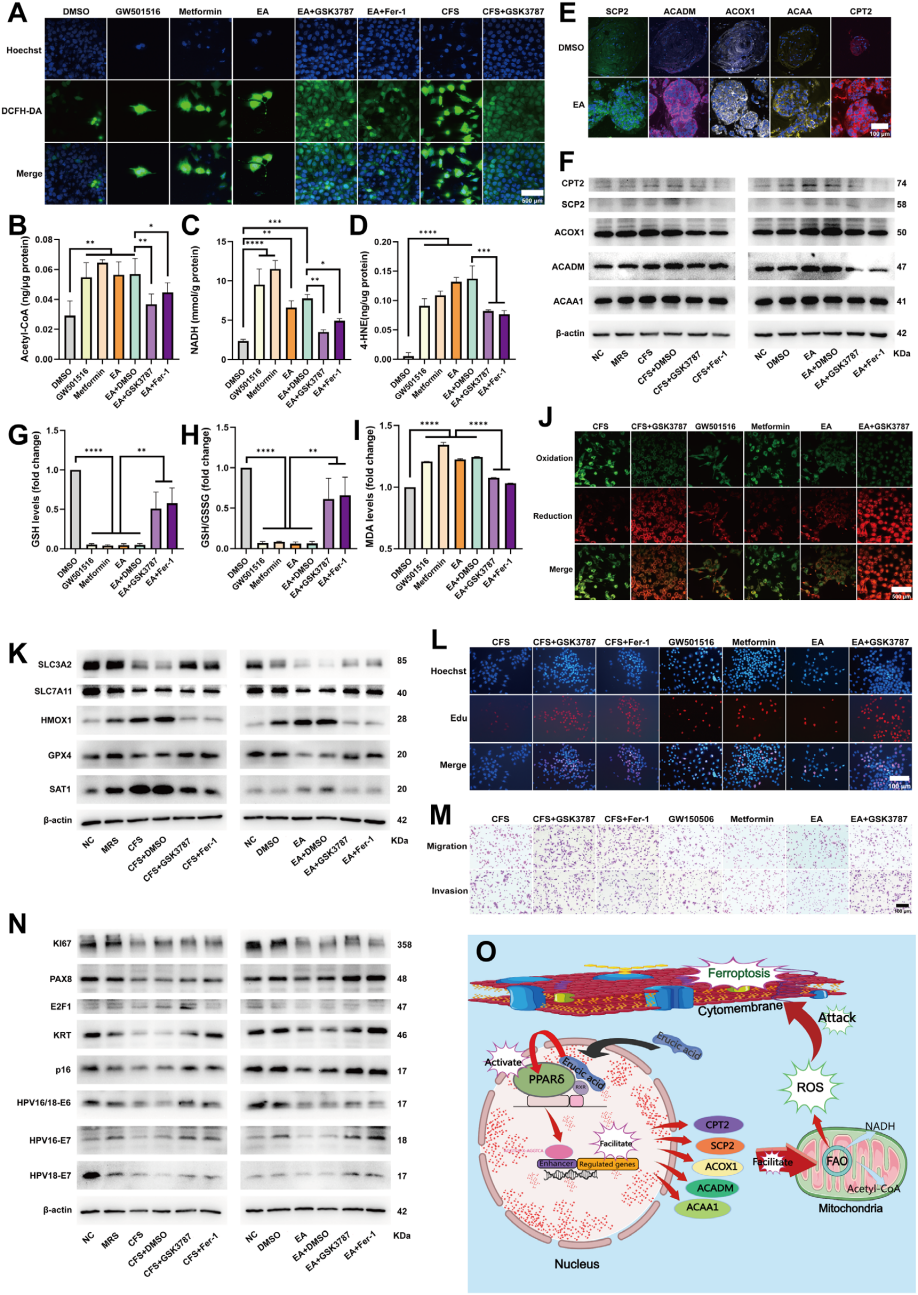
Erucic acid induces ferroptosis via PPAR‐δ pathway. A) ROS assays of Ca Ski cells treated with DMSO, GW501516 (a PPARδ receptor agonists), Metformin (a promoter of fatty acid oxidation), EA, EA+DMSO, EA+GSK3787 (GSK3787 is a PPARδ receptor antagonist), EA+Fer‐1, CFS and CFS+ GSK3787. Blue fluorescence: Hoechst (nuclei of all the cells). Green fluorescence: ROS. Scale bars, 500 µm. B) Bar chart of the Acetyl‐CoA levels. Statistical analyses: paired t tests, ns *p >* 0.05, *p < 0.05, **p < 0.01. C) Bar chart of the NADH levels. Statistical analyses: paired t tests, *p < 0.05, **p < 0.01, ***p < 0.001, ****p < 0.0001. D) Bar chart of the levels of 4‐HNE. Statistical analyses: paired t tests, ***p < 0.001, ****p < 0.0001. E) Immunohistofluorescence staining results relating to FAO of cervical organoids treated with DMSO and EA. Scale bars, 100 µm. F) Western Blot results relating to FAO of Ca Ski cells treated with 10%PBS, 10%MRS, 10%CFS, 10%CFS+DMSO, 10%CFS+GSK3787, 10%CFS+Fer‐1, Blank, DMSO, EA, EA+DMSO, EA+ GSK3787, EA+Fer‐1. G) Bar chart of the GSH levels (fold change). Statistical analyses: paired t tests, ns *p >* 0.05, **p < 0.01, ****p < 0.0001. H) Bar chart of the ratio of GSH and GSSG (fold change). Statistical analyses: paired t tests, ns *p >* 0.05, **p < 0.01, ****p < 0.0001. I) Bar chart of the levels of MDA. Statistical analyses: paired t tests, ****p < 0.0001. J) C11‐Bodipy assays of Ca Ski cells treated with CFS, CFS+GSK3787, GW501516, Metformin, EA and EA+ GSK3787. Green fluorescence: oxidation. Red fluorescence: reduction. Scale bars, 500 µm. K) Western Blot results relating to ferroptosis of Ca Ski cells treated with 10%PBS, 10%MRS, 10%CFS, 10%CFS+DMSO, 10%CFS+GSK3787, 10%CFS+Fer‐1, Blank, DMSO, EA, EA+DMSO, EA+ GSK3787, EA+Fer‐1. L) Edu assays of Ca Ski cells treated with 10%CFS, 10%CFS+GSK3787, 10%CFS+Fer‐1, GW501516, Metformin, EA and EA+ GSK3787. Blue fluorescence: Hoechst (nuclei of all the cells). Red fluorescence: 5‐Ethynyl‐2′‐deoxyuridine (proliferating cells). Scale bars, 100 µm. M) Transwell assays of Ca Ski cells treated with 10%CFS, 10%CFS+GSK3787, 10%CFS+Fer‐1, GW501516, Metformin, EA and EA+ GSK3787. Scale bars, 100 µm. N) Western Blot results relating to proliferation and HPV of Ca Ski cells treated with 10%PBS, 10%MRS, 10%CFS, 10%CFS+DMSO, 10%CFS+GSK3787, 10%CFS+Fer‐1, Blank, DMSO, EA, EA+DMSO, EA+ GSK3787, EA+Fer‐1. O) Mechanistic diagram of the process of EA‐induced ferroptosis. Made by Medpeer.

In view of the above, we speculated that EA activated the PPAR‐δ pathway, exacerbating downstream FAO and producing excessive ROS, which in turn led to ferroptosis. In order to verify the above hypothesis, we detected the ROS levels by DCFH‐DA in Ca Ski cells treated with DMSO, GW501516 (a PPARδ receptor agonists), Metformin (1,1‐Dimethylbiguanide hydrochloride, a promoter of fatty acid oxidation), EA, EA+DMSO, EA+GSK3787 (GSK3787 is a PPARδ receptor antagonist) and EA+Fer‐1. The results showed that the ROS levels of Ca Ski cells treated with GW501516, Metformin, EA and EA+DMSO increased significantly, and the increasing trend could be partially rescued by GSK3787 and Fer‐1 (**Figure** **6**A). In addition, the ROS levels of cells treated with CFS also increased, and it could also be rescued by GSK3787 and Fer‐1 (Figures 6A and 4J). On the other hand, the levels of Acetyl‐CoA and NADH, the FAO products, were also significantly increased in Ca Ski cells treated with GW501516, Metformin, EA and EA+DMSO, and the increasing trend could be rescued by GSK3787 and Fer‐1 (Figure 6B,C). The enzymes related to FAO in the downstream of PPAR‐δ pathway include SCP2 (sterol carrier protein 2), ACADM (acyl‐CoA dehydrogenase), ACOX1 (acyl‐CoA oxidase), ACAA1 (acetyl‐CoA acyltransferase 1) and CPT2 (carnitine O‐palmitoyltransferase 2). ACOX1 is the initial enzyme in the peroxisomal fatty acid beta‐oxidation pathway, catalyzing the oxidative dehydrogenation of long‐chain and very‐long‐chain fatty acids to produce H_2_O_2_ (a type of ROS). We detected the protein levels of the above enzymes in EA‐treated organoids and found that their expression levels were significantly increased (Figure 6E). Moreover, Western Blot experiments were performed in Ca Ski cells to detect the protein levels of the above FAO‐related enzymes again, and it was found that both CFS and EA could upregulate their levels, and the results could be rescued by GSK3787 and Fer‐1 (Figure 6F and Figure S5A–E, Supporting Information). The above results indicated that EA indeed enhanced FAO by activating the PPAR‐δ pathway and also increased the level of ROS at the same time. Metformin can activate signaling pathways such as LKB1‐AMPK (AMP‐activated protein kinase), including downstream acetyl‐CoA carboxylase, thereby inhibiting hepatic gluconeogenesis and promoting fatty acid oxidation. Following Metformin treatment, ROS levels in Ca Ski cells also showed a significant increase, demonstrating that promoting fatty acid oxidation indeed elevated ROS levels.

To explore the relationship between FAO and ferroptosis, we detected the levels of GSH, GSSG, 4‐HNE and MDA in Ca Ski cells treated with DMSO, GW501516 (a PPARδ receptor agonists), Metformin (a promoter of fatty acid oxidation), EA, EA+DMSO, EA+GSK3787 (GSK3787 is a PPARδ receptor antagonist) and EA+Fer‐1. Compared with the results in Figure 5G‐J, increased levels of MDA and HNE and depletion of glutathione were also observed in cells treated with GW501516 and Metformin, and the above results could be rescued by GSK3787 (Figure 6D, G, H,I). In C11‐Bodipy assays, it was observed that ferroptosis was aggravated in cells treated with CFS, GW501516, Metformin, and EA, and the results could be rescued by GSK3787 (Figure 6J). The results of Western Blot revealed that CFS/EA treatment induced ferroptosis, characterized by upregulation of pro‐ferroptotic markers (HMOX1, SAT1) and downregulation of anti‐ferroptotic factors (SLC3A2, SLC7A11, GPX4) (Figure 6K and Figure S5F–J, Supporting Information). The above experimental findings indicated that activation of the PPAR‐δ pathway mediated EA‐induced ferroptosis. Moreover, the use of Metformin to directly promote FAO can also induce ferroptosis.

The above experimental results demonstrated that EA exacerbated FAO (fatty acid oxidation) by activating the PPAR‐δ pathway, while simultaneously increasing ROS levels, thereby inducing ferroptosis in cervical cancer. Edu and transwell assays confirmed that blocking the PPAR‐δ pathway with GSK3787 can rescue the reducing effects of CFS and EA on the proliferation, migration, and invasion capabilities of cervical cancer (Figure 6L,M). The Western Blot results demonstrated that GSK3787, which blocked the PPAR‐δ pathway, can restore the elevation of molecules related to cancer proliferation and HPV viral load, further validating that PPAR‐δ blockade reversed the inhibitory effect of EA on cervical cancer (Figure 6N and Figure S5K–R, Supporting Information).

Conclusively, we found that EA could bind to and activate PPAR‐δ receptors, leading to increased expression of molecules such as ACOX1 and exacerbation of FAO, which resulted in massive ROS generation. Excessive ROS attacked cytomembranes, triggering ferroptosis (Figure 6O).

## 3 Discussion

The vaginal microbiome, as an important component of the female reproductive system, has received little attention from scholars. This has resulted in the cervical cancer microbial research, up to the present, remaining superficial, with very few studies truly elucidating the mechanisms of interaction between the two. Our study breakthrough provides the first in‐depth elucidation of the specific mechanism by which *Lactobacillus crispatus*—the dominant species in healthy vaginal microbiota—influences cervical cancer. Novel findings reveal that EA, a metabolite of *Lactobacillus crispatus*, exacerbates fatty acid oxidation (FAO) by activating the PPAR‐δ pathway, releasing substantial reactive oxygen species (ROS) that subsequently induce ferroptosis in cancer cells. Furthermore, the research employs cervical cancer organoids as a model for single‐cell sequencing and ferroptosis validation, thereby enhancing the credibility of the experimental results. Our study fills the gap in this research field and provides new insights for future studies.

The microorganisms, as a kind of environmental factors, that colonize the human body, can shift the metabolism, growth pattern and function of neoplastic cells and shape the tumor microenvironment.^[^ ^21^ ^]^ Among HPV‐positive patients, *Gardnerella vaginalis, Enterococcus spp., Staphylococcus spp., Proteus spp., and Atopobium* are frequently identified. The altered vaginal microbiota may contribute to HPV cervical infection, progression, and clearance. For a long time, a healthy vaginal microbiota has been considered to play an important role in the vaginal environment. One of its key functions is maintaining a relatively acidic environment and producing protective proteins, serving as the first line of defense against pathogens. Specifically, the vaginal microbiota dominated by *Lactobacilli* creates biofilms and barriers by adhering to the mucus, competing with and preventing the colonization of pathogens. Additionally, they produce protective antimicrobial compounds, including bacteriocin‐like substances, biosurfactants, hydrogen peroxide and lactic acid.^[^ ^22^, ^23^, ^24^, ^25^ ^]^ However, some other researches indicating that the vaginal microbiota can secrete bacterial genotoxins, leading to the development of chronic inflammation of mucosal epithelium, creating a favorable environment for the development of cancer, including inducing cellular and DNA damage.^[^ ^26^, ^27^, ^28^ ^]^ Additionally, the microbiota‐derived metabolites have a significant relationship with immune responses and inflammation, which in turn directly or indirectly influence the development and progression of tumors.^[^ ^29^, ^30^, ^31^ ^]^ Thus, it can be seen that previous studies on vaginal microbiota have been relatively superficial and failed to thoroughly investigate the specific mechanisms by which vaginal microbial communities influence the cervix, whereas this research fills that gap.

Erucic Acid is a monounsaturated omega‐9 fatty acid with the chemical name cis‐13‐docosenoic acid and a molecular formula of C_22_H_42_O_2_.^[^ ^32^ ^]^ Erucic acid can also be prepared from the seeds of Brassicaceae plants, making its production advantageous due to the wide availability of raw materials. Previous studies have shown that erucic acid participates in various biological processes and exhibits antitumor, neuroprotective, and anti‐inflammatory effects in diseases such as breast cancer, colorectal cancer, glioblastoma and neuroblastoma.^[^ ^32^, ^33^ ^]^ At clinically achievable concentrations, erucic acid reduces DNA synthesis in C6 rat glioblastoma, indicating its potential antitumor activity.^[^ ^34^ ^]^ In colorectal cancer, it enhances the antitumor efficacy of cisplatin by elevating ROS levels, stimulating TRPM2, depleting glutathione and inducing mitochondrial dysfunction.^[^ ^35^ ^]^ Additionally, erucic acid promotes ferroptosis by inhibiting the phosphorylation of transcription activator 3, ultimately suppressing the effector functions of CD8+ T cells, thereby achieving therapeutic effects in systemic lupus erythematosus.^[^ ^36^ ^]^ These findings demonstrate that erucic acid exhibits antitumor properties, elevates ROS levels and induces ferroptosis in various diseases.

In our research, it was also found that erucic acid can lead to increased levels of ROS in cancer cells and ultimately induce ferroptosis. To further investigate its mechanism, we explored the KEGG database and discovered that erucic acid acts as a ligand to activate the nuclear receptor PPARδ (peroxisome proliferator‐activated receptor delta), subsequently triggering multiple downstream biological processes including lipid synthesis, cholesterol metabolism, fatty acid transport and fatty acid oxidation. These processes play crucial roles in regulating lipid metabolism, energy homeostasis and inflammatory responses. In breast cancer, PPARβ/δ agonists inhibit the invasion of cancer cells by blocking ANGPTL4 transcription.^[^ ^37^ ^]^ Among colorectal cancer patients, those with higher PPARβ/δ expression exhibit significantly higher survival rates.^[^ ^38^ ^]^ Research in melanoma demonstrates that ligand activation of PPARβ/δ suppresses tumor cell proliferation.^[^ ^39^ ^]^ In neuroblastoma, ligand‐mediated activation of PPARβ/δ promotes cell differentiation and inhibits proliferation.^[^ ^40^ ^]^ For pancreatic cancer, ligand‐mediated activation of PPARβ/δ inhibits cytokine‐induced invasion and migration.^[^ ^41^ ^]^ PPARδ is a target of the tumor suppressor APC in colorectal cancer cells and is highly expressed in the cancer cells which possess inactivation mutations of APC. β‐catenin/Tcf‐4, the transcription factors in the APC signaling pathway, can interact with and activate the promotor of PPARδ directly.^[^ ^42^, ^43^, ^44^ ^]^ Although the role of PPARδ in regulating cell metabolism and inflammation has been extensively studied, research on the direct connection between PPARδ and ferroptosis remains relatively limited. Notably, PPARα, another member of the PPAR family, has been shown to be associated with ferroptosis.^[^ ^45^ ^]^ Studies have shown that PPARα can inhibit ferroptosis by promoting the expression of glutathione peroxidase 4 (GPX4) and inhibiting the expression of the transferrin (TRF). PPARα directly induces the expression of GPX4 by binding to the PPRE (PPAR response element) in intron 3 of the GPX4. Additionally, PPARα‐knockout mice exhibited much more iron accumulation and ferroptosis in the liver compared to wild‐type mice under a high‐iron diet.^[^ ^46^ ^]^ These studies suggest that activation of PPARα can alleviate iron overload‐induced ferroptosis in the liver of mice through GPX4 and TRF, indicating that PPARα may be a promising therapeutic target for drug discovery of ferroptosis.^[^ ^47^, ^48^ ^]^ Although the direct relationship between PPARδ and ferroptosis is not yet clear, considering the similarity and functional redundancy among members of the PPAR family, PPARδ may also participate in regulating ferroptosis through a similar mechanism. More studies are needed to further explore the role of PPARδ in ferroptosis and its potential molecular mechanisms.

In our study, it was found that after activation of PPARδ receptor by erucic acid, the extent of downstream FAO was exacerbated by upregulating CPT2, SCP2, ACOX1, ACADM and ACAA1, who facilitated the transport of fatty acids to the mitochondria and enhance the efficiency of peroxisomal beta‐oxidation. ACOX1 is the initial enzyme in the peroxisomal fatty acid beta‐oxidation pathway, catalyzing the oxidative dehydrogenation of long‐chain and very‐long‐chain fatty acids to produce H_2_O_2_ (a type of ROS). The primary endogenous source of ROS is electron leakage from the mitochondrial respiratory chain. Within the ETC (electron transport chain), complexes I and III directly transfer electrons to oxygen, generating O_2_^_^ —a form of ROS. FAO produces substantial NADH, which fuels the operation of the ETC and exacerbates electron leakage and ROS generation.^[^ ^49^ ^]^ Tumor cells express high levels of antioxidant proteins to prevent ROS accumulation and avoid ROS‐driven ferroptosis.^[^ ^50^ ^]^ However, excessively enhanced FAO produces surplus ROS, overwhelming the tumor cells' control over ROS levels and thereby inducing ferroptosis in cancer cells.^[^ ^51^, ^52^, ^53^ ^]^ The treatment method of killing tumors by increasing ROS has been applied for many years, such as photodynamic therapy. However, the clinical application scope of this exogenous stimulation approach for ROS enhancement remains limited. Currently, research on increasing ROS through endogenous methods to kill tumors is relatively scarce, but an increasing number of scholars are dedicated to this field. For example, Mingxi Qiao et al. developed a self‐assembled nanocomplex called Ato, which activates AMPK to promote highly suppressed mitochondrial FAO in cancer cells, thereby generating ROS. The ROS produced by FAO further activates AMPK, establishing a positive feedback loop that sustains continuous ROS production, ultimately achieving tumor‐killing effects.^[^ ^54^ ^]^ Metformin, as a FAO promotor by activating LKB1‐AMPK and acetyl‐CoA carboxylase, has already been confirmed by Jingjing Yang et al. that could induce ferroptosis and suppress breast cancer growth by inhibiting UFMylation of SLC7A11 and increasing intracellular ROS levels.^[^ ^55^ ^]^ Hence, activation of FAO indeed induces ferroptosis.

A recent study by Meilin et al. says that unsaturated long‐chain fatty acids like oleic acid can simultaneously inhibit *L. iners* and BV‐associated bacteria while promoting growth of non‐iners lactobacilli, promoting a more health‐associated microbiome composition in an in vitro model.^[^ ^56^ ^]^ Since the study by Meilin et al. primarily focused on bacterial vaginosis, the emphasis was placed on the effects of metabolites such as oleic acid on other bacteria. However, in our study, the metabolites of *L. crispatus*, erucic acid, which is also an unsaturated long‐chain fatty acid, has been experimentally proven to promote ferroptosis in cancer cells, thereby inhibiting tumor development and progression. Therefore, we speculate that oleic acid and erucic acid, as similar metabolites, may both inhibit the proliferation of other bacteria and affect host cells. We believe that our researches’ direction and conclusions provide complementary insights, while also broadening the scope and perspective, which is of great significance and deserves further study in the future.

We successfully establish organoids of cervical cancer infected with different types of HPV, including HPV16 and HPV 82, and the experimental results obtained from these organoids are similar, indicating that our conclusions are universally applicable. We believe this point is extremely important because, although HPV16 is the most significant infection type associated with cervical cancer, there are actually many other types of HPV infections present in clinical settings. The models commonly used in laboratories are predominantly cell lines infected with HPV16 or HPV18, lacking infection with medium‐risk or other types of HPV. Over‐focusing on specific high‐risk HPV (such as HPV16 and HPV18) while neglecting other types of HPV has been prevalent for a long time. This imbalanced research emphasis may lead to inadequate understanding of the complexity and diversity of HPV infections, which is clearly insufficient for experimental research. Our HPV82 organoids, however, effectively address this shortcoming. Moreover, organoids are often more cost‐effective and, in some instances, more physiologically relevant than patient‐derived tumor xenograft animal models, depending on the experimental design.

Another notable point is that, apart from co‐culture methods, current technologies are not sufficient to cultivate organoids containing immune cells, a fact that is also confirmed in our single‐cell sequencing. Although we identified two distinct subpopulations of cells that co‐expressed inflammatory and tumor‐related markers, confirming the propensity of organoids toward inflammation, we ultimately classified these cells as tumor cells because we did not co‐culture them with immune cells. We believe that the appearance of subpopulations of cells with inflammatory markers in single‐cell sequencing is due to the self‐constructive function of the organoids themselves and the stemness of the tumor cells. Although organoids lacking immune cells pose limitations when studying immune‐related topics, they also ensure the stability of the organoid models. There are already many scholars researching models of co‐culturing organoids with immune cells or vascular cells. We believe this is not a difficult problem, and even in the near future, organoid culture methods will compensate for the shortcomings of immune and vascular cells, thereby reaching a higher level.^[^ ^57^, ^58^, ^59^ ^]^

Erucic acid as a ligand activating PPAR‐δ has been extensively documented in the literature. Our study confirmed that EA activation of PPAR‐δ not only exacerbated FAO but also elevated ROS level, leading to ferroptosis, all of which can be rescued by PPAR‐δ receptor antagonists. The chain reaction caused by ROS attacking polyunsaturated fatty acids on cell membranes, resulting in lipid peroxidation accumulation, has been widely recognized by researchers as the primary mechanism of ferroptosis.^[^ ^60^ ^]^ Thus, we have demonstrated a novel finding: erucic acid, a metabolite of *Lactobacillus crispatus*, can induce ferroptosis in cervical cancer through the PPAR‐δ pathway. Regarding ferroptosis and signaling pathways, there may be other signaling pathways involved in this study, but the PPARδ pathway is the most significant. The roles of erucic acid and PPARδ in lipid metabolism have been identified as potential therapeutic targets for various diseases, making this field one of the prominent research hotspots.^[^ ^61^ ^]^ In breast cancer, erucic acid has been demonstrated to combine with phytosphingosine to create cationic nanoemulsions for plasmid delivery, thereby exerting anticancer effects.^[^ ^62^ ^]^ In lung cancer, erucic acid has been shown to form liposomes with oleic acid, another monounsaturated fatty acid, encapsulating various anticancer drugs to achieve therapeutic outcomes.^[^ ^63^ ^]^ These findings highlight the broad feasibility of erucic acid in clinical practical applications. Given erucic acid's promoting effect on lipid metabolism and its potential to induce cardiovascular blockages and related diseases, the use of erucic acid in cervical cancer treatment may be more appropriate through direct vaginal administration rather than intravenous injection into the bloodstream. Therefore, further clinical attempts are necessary for erucic acid's practical medical applications (**Table** **1**).

**Table 1:** Key Resources Table.

Reagent or Resource	Source	Identifier
**Antibodies**		
Rabbit polyclonal anti‐SLC3A2	Proteintech	Cat# 15193‐1‐AP , RRID: AB_2254909
Rabbit polyclonal anti‐SLC7A11	Proteintech	Cat# 26864‐1‐AP , RRID: AB_2880661
Rabbit polyclonal anti‐HMOX1	Proteintech	Cat# 10701‐1‐AP , RRID: AB_2118685
Mouse monoclonal anti‐GPX4	Proteintech	Cat# 67763‐1‐Ig , RRID: AB_2909469
Rabbit polyclonal anti‐SAT1	Proteintech	Cat# 10708‐1‐AP , RRID: AB_2877739
Rabbit monoclonal anti‐CPT2(EPR13626)	Abcam	Cat# AB181114 , RRID: AB_2687503
Rabbit polyclonal anti‐Sterol carrier protein 2	Proteintech	Cat# 23006‐1‐AP, RRID: 23006‐1‐AP
Rabbit monoclonal anti‐ACOX1(EPR19038)	Abcam	Cat# AB184032 , RRID: AB_2904240
Rabbit monoclonal anti‐ACADM(EPR3708)	Abcam	Cat# AB92461 , RRID: AB_10563530
Rabbit polyclonal anti‐ACAA1	Proteintech	Cat# 12319‐2‐AP , RRID:2289045
Mouse monoclonal anti‐Beta Actin	Proteintech	Cat# 66009‐1‐Ig , RRID: AB_2687938
Rabbit monoclonal anti‐Ki67(SP6)	Abcam	Cat# AB16667 , RRID: AB_302459
Rabbit polyclonal anti‐PAX8	Proteintech	Cat# 10336‐1‐AP , RRID: AB_2236705
Rabbit monoclonal anti‐E2F‐1	Cell Signaling Technology	Cat# 3742 , RRID: AB_2096936
Mouse monoclonal anti‐Pan‐Keratin(C11)	Cell Signaling Technology	Cat# 4545 , RRID: AB_490860
Rabbit monoclonal anti‐p16INK4a(EPR1473)	Abcam	Cat# AB108349 , RRID: AB_10858268
Mouse monoclonal anti‐HPV Type 16/18 E6(C1P5)	Thermo Fisher Scientific	Cat# MA1‐46057 , RRID: AB_1017663
Mouse monoclonal anti‐HPV Type 16‐E7(8C9)	Thermo Fisher Scientific	Cat# 28‐0006 , RRID: AB_86768
Mouse monoclonal anti‐HPV Type 18‐E7(GT881)	Abcam	Cat# MA5‐35923 , RRID: AB_2866540
Goat anti‐Rabbit IgG(H+L) HRP‐conjugated Secondary Antibody	Proteintech	Cat# SA00001‐2 , RRID: AB_2722564
Goat anti‐Mouse IgG(H+L) HRP‐conjugated Secondary Antibody	Proteintech	Cat# SA00001‐1 , RRID: AB_2722565
**Bacterial and virus strains**		
Lactobacillus crispatus strain 5578	This study	N/A
LV16‐GFP‐Homo‐198	GenePharma	N/A
**Biological samples**		
Human vaginal secretions	This study	N/A
Human cervical cancer tissue	This study	N/A
PDO of cervical cancer	This study	N/A
**Chemicals, peptides, and recombinant proteins**		
GSK 3787	Selleck	Cat# 188591‐46‐0
GW501516	MedChemExpress	Cat# HY‐10838
Metformin	MedChemExpress	Cat#
Erucic acid	MedChemExpress	Cat# HY‐N7109
Ferrostatin‐1	MedChemExpress	Cat# HY‐100579
Matrigel	Mogengel Biotechnology	Cat# 082703
FBS	VivaCell Biosciences	Cat# C04001‐500
RPMI 1640 Medium	Macgene	Cat# CM10040
MEM/EBSS L‐glutamine	Macgene	Cat# CM10010
DMEM/F12	Macgene	Cat# CM10092
LB Broth	Solarbio	Cat# L8291
MRS Agar	Solarbio	Cat# M8330
Lactobacilli MRS Broth	ELITE Biotech	Cat# M264‐01
R‐spondin 1 (RSPO1)	made in‐house	N/A
GlutaMax	Thermo Fisher	Cat# 35050‐038
HEPES	Thermo Fisher	Cat# 15630‐056
Noggin‐Fc fusion protein	U‐Protein Express	Cat# N002‐100 ml
FGF2	PeproTech	Cat# 100–18B
FGF10	PeproTech	Cat# 100–26
EGF	PeproTech	Cat# AF‐100‐15
A83‐01	Tocris	Cat# 2939
ROCK inhibitor (Y‐27632)	Abmole	Cat# Y‐27632
Forskolin	Bio‐Techne	Cat# 1099
B27 supplement	Life Technologies	Cat# 17504‐044
N‐acetyl‐L‐cysteine	Sigma‐Aldrich	Cat# A9165
Nicotinamide	Sigma‐Aldrich	Cat# N0636
Primocin	InvivoGen	Cat# Ant‐pm‐1
Prostaglandin E2	Tocris	Cat# 2296
CHIR (Chir99021)	Sigma‐Aldrich	Cat# SML1046
Penicillin‐Streptomycin	Meilunbio	Cat# MA0110‐100
TrypLE Express	GIBCO	Cat# 12605‐010
2‐Mercaptoethanol	GIBCO	Cat# 21985‐023‐50
Roche complete‐EDTA free protease inhibitor tablets	Millipore‐Sigma	Cat# 05056489001
Hybri‐max DMSO	Millipore‐Sigma	Cat# D2650
PEG300	Solarbio	Cat# IP9020
DAPI	Beyotime	Cat# C1002
Hoechst 33342	RIBOBIO	Cat# C10310‐1
Phalloidin‐AF555	Cohesion Biosciences	Cat# CRG1034
Triton X‐100	Solarbio	Cat# P1080
Crystal Violet	Beyotime	Cat# C0121‐500
Tween‐80	Solarbio	Cat# T8360
Cole's Hematoxylin Solution (For Conventional Stain)	Solarbio	Cat# G1140
Antigen Retrieval buffer(50×) Citrate buffer of pH6.0	ImmunoWay	Cat# YS0002
Antigen Retrieval buffer(50×) Tris‐EDTA of pH9.0	ImmunoWay	Cat# YS0004
Primary Tissue Storage Solution	BioGenous	Cat# K601005
Tumor Tissue Digestion Solution	BioGenous	Cat# K601003
Anti‐Adherence Rinsing Solution	BioGenous	Cat# E238002
Eosin dye	Nanjing Jiancheng Bioengineering Institute	Cat# D019
RIPA Lysis Buffer	Meilunbio	Cat# MA0151
Blot Blocking Buffer	NCM Biotech	Cat# P30500
ABScript III RT Master Mix for qPCR	Abclonal	Cat# RK20428
2× Universal SYBR Green Fast qPCR Mix	Abclonal	Cat# RK21203
**Critical commercial assays**		
Cell Counting Kit‐8	Meilunbio	Cat# MA0218‐5‐Feb‐29J
Cell‐Light EdU Apollo567 In Vitro Kit	RIBOBIO	Cat# C10310‐1
Organoid Viability ATP Assay Kit	BioGenous	Cat# E238003
Live/dead bacteria dual staining kit	BIOESN	Cat# BES2066CDD
AO/PI Mixed Dye	MONWEI	Cat# SC1009
Reactive Oxygen Species Assay Kit	Beyotime	Cat# S0033M
C11‐BODIPY 581/591 Lipid Peroxidation Sensor	Maokang Biotechnology	Cat# MX5211‐1MG
RNAprep Pure Micro Kit	TIANGEN	Cat# DP420
Enhanced BCA Protein Assay Kit	Beyotime	Cat# P0009
4‐HNE (4‐Hydroxynonenal) ELISA Kit	Elabscience	Cat# E‐EL‐0128
A‐CoA (Acetyl Coenzyme A) ELISA Kit	Elabscience	Cat# E‐EL‐0125
Fatty Acid Oxidation (FAO) Colorimetric Assay Kit	Elabscience	Cat# E‐BC‐K784‐M
GSH and GSSG Assay Kit	Beyotime	Cat# S0053
Lipid Peroxidation MDA Assay Kit	Beyotime	Cat# S0131M
**Deposited data**		
ScRNA sequencing	This paper; SRA	Public when published.
16S rRNA sequencing	This paper; SRA	Public when published.
Untargeted metabolomics	This paper; SRA	Public when published.
**Experimental models: cell lines**		
Ca Ski	ATCC	CRM‐CRL‐1550
C‐33A	ATCC	HTB‐31
**Experimental models: Organisms/strains**		
Mouse: BALB/cAnN‐Foxn1nu/nu/Rj	Charles River Laboratories	RRID: IMSR_RJ: BALB‐C‐NUDE
**Software and algorithms**		
10× Genomics Cell Ranger (v7.2.0)	10× Genomics	https://support.10×genomics.com/single‐cell‐gene‐expression/software/overview/welcome; RRID:SCR_017344
R package Seurat (v4.1.1)	Comprehensive R Archive Network	https://satijalab.org/seurat/; RRID: SCR_016341
R (v4.1.2)	R Core Team	https://www.r‐project.org/; RRID: SCR_001905
RStudio (v4.3.3)	R Studio Team	https://www.rstudio.com/; RRID: SCR_000432
GraphPad Prism (v9.0)	GraphPad Software	https://www.graphpad.com/; RRID:SCR_002798
ImageJ (v1.54f)	National Institutes of Health	https://imagej.org; RRID:SCR_003070
Python (v3.6.13)	Python Programming Language	https://www.python.org/; RRID:SCR_008394
Cutadapt (v3.3)	Cutadapt	https://bio.tools/cutadapt; RRID:SCR_011841
Flash (v1.2.11)	Flash Software	http://ccb.jhu.edu/software/FLASH/; RRID:SCR_005531
Fastp (v0.23.1)	National Science Foundation of China	https://github.com/OpenGene/fastp; RRID:SCR_016962
Vsearch (v2.16.0)	VSEARCH	https://github.com/torognes/vsearch; RRID:SCR_024494
Uparse (v7.0)	UPARSE	http://drive5.com/uparse/; RRID:SCR_005020
Muscle (v 3.8.1551)	MUSCLE	http://www.ebi.ac.uk/Tools/msa/muscle/; RRID:SCR_011812
Qiime (qiime1.9.1)	QIIME	http://qiime.org/; RRID:SCR_008249
Perl (v5.26.2)	Perl Programming Language	http://www.perl.org; RRID:SCR_018313
Medpeer	Medpeer	http://www.medpeer.cn/
MassLynx (v4.2)	MassLynx	http://www.waters.com/waters/en_US/MassLynx‐MS‐Software/nav.htm?cid=513662&locale=en_US RRID:SCR_014271
Progenesis QI	Progenesis QI software	http://www.nonlinear.com/progenesis/qi‐for‐proteomics/;RRID:SCR_018923

## 4 Experimental Section

### Cell Derived Xenograft

For drug treatment models (Figures 6C), Ca Ski cells (5.0 × 10^6^ cells in 100 µL saline solution) were injected subcutaneously into the left dorsal flank of 4‐ to 6‐week‐old female BALB/cAnN‐Foxn1nu/nu/Rj (RRID: IMSR_RJ: BALB‐C‐NUDE). Mice were randomly divided into 4 groups (MRS versus CFS; DMSO versus EA) after the establishment of tumors. MRS and CFS (both diluted to a 10% concentration with saline solution) were administered twice a week, DMSO and EA (10 mm) were treated twice a week. The stock solution of EA (100 mm) was formulated in 10% DMSO, 40% PEG300, 5% Tween 80 and 45% saline solution. The equal volume of DMSO was dissolved using the dissolution process of EA. Four kinds of solution were given to mice by intratumoral injection and the injection volume were all 10 µL per mouse. During intratumoral injection, the z‐shaped insertion method was used, and the drug was injected from two opposite insertion points. After injection, it was necessary to withdraw the needle slowly to minimize drug leakage. Tumor volume was measured with a digital caliper, and tumor volume (mm^3^) was calculated using the following formula: 1/2axb^2^, where a and b were the longest and shortest diameter in mm, respectively. At the end point, tumors were harvested and weighed. Tumor tissues were stored in −80 °C or fixed in 10% formalin. All procedures were approved by the Animal Experimentation Ethics Committee of the Qilu Hospital of Shandong University (Approval number: DWLL‐2024‐327).

### Patient Derived Organoid Xenograft

PDO‐HPV16‐a and PDO‐HPV82 were scraped by the pipette tips along with the matrigel from the bottom of the culture dish. After centrifugation at 300 ×g for 5 min, organoids were resuspended with saline solution. Then repeatedly aspirated and dispensed the sample until they were fully mechanically digested into single‐cell suspension using insulin needles with the diameter of 0.3 mm. 100 µL of the mixture was injected into the subcutaneous of the right forelimb of 4‐ to 6‐week‐old female BALB/cAnN‐Foxn1nu/nu/Rj (RRID: IMSR_RJ: BALB‐C‐NUDE) with a 1 mL sterile syringe (about 3.0 × 10^6^ cells per mice). The rate of tumor formation differed from CDX and we routinely monitored the tumor size. However, even after 3 months of injection, the tumor volume was still far below the minimum requirement of 100 mm^3^. Nearly half of the tumors could no longer be palpated significantly. Therefore, unfortunately, we were unable to proceed with the drug experiments using the PDOX model. All procedures were approved by the Animal Experimentation Ethics Committee of the Qilu Hospital of Shandong University (Approval number: DWLL‐2024‐327).

### Vaginal Secretions Sample Collection

From June 2023 to June 2024, vaginal secretions samples were collected from healthy cervix patients and cervical cancer patients in the Qilu Hospital of Shandong University. Inclusion criteria and exclusion criteria are showed in Table S2, Supporting Information. Cohort demographics are showed in Table S1, Supporting Information. All volunteers were patients admitted to the Department of Obstetrics and Gynecology in Qilu Hospital of Shandong University for surgical treatment due to diseases. Before the patients accepting surgeries and antibiotic treatments, the researchers decided whether to include the patients in the study basing on the information in the admission records. After meeting the criteria and obtaining informed consents, the gynecologists conducted professional gynecological examinations on the patients in the gynecology examination room and collected vaginal secretions using specialized collection swabs and storage tubes. A few of samples were used to isolation of *L. crispatus* in Microbiology Teaching and Research Section, School of Basic Medical Sciences, Shandong University, and most samples were stored at −80 °C until the 16S rRNA sequencing. All procedures were approved by the Ethical Committee of School of Basic Medical Sciences, Shandong University (Approval number: ECSBMSSDU2024‐1‐159).

### Cervical Cancer Tissue Sample Collection

From September 2023 to December 2023, cervical cancer tissue sample were collected in Qilu Hospital of Shandong University. Inclusion criteria and exclusion criteria are showed in Table S1, Supporting Information. All volunteers were patients admitted to the Department of Obstetrics and Gynecology in Qilu Hospital of Shandong University for surgical treatment due to cervical cancer. Before the patients accepting treatments, the researchers decided whether to include the patients in the study basing on the information in the admission records and gynecological examinations before surgeries. After obtaining informed consents of patients, the researchers removed a piece of tumor tissue whose size was similar to a soybean under the assistance of the surgical doctors and operating room nurses. All procedures were approved by the Research Ethical Committee of Qilu Hospital of Shandong University (Approval number: KLYY‐202309‐055). All procedures had passed the scientific review by the Research Department of Qilu Hospital, Shandong University (Approval number: QLCR20230484).

### Cell Lines

Ca Ski cells and C‐33A cells were cultured in 1x RPMI1640 and 1x MEM, respectively with 10% FBS and 1% Penicillin/Streptomycin at 37 °C and 5% CO2. Both Ca Ski cells (CRM‐CRL‐1550) and C‐33A(HTB‐31) cells were ordered from ATCC.

### PDO

During the transportation of samples from the operating room to the laboratory, the cervical cancer tissues were stored in Primary Tissue Storage Solution at 37 °C. The tissues were mechanically minced using scalpels until the fragments can be easily aspirated and dispensed with 1 ml pipette tips without clogging, followed by digestion in Tumor Tissue Digestion Solution (BioGenous) for 40 min in the 37 °C shaker. The resulting cells were filtered using a 100 µm cell filter. The resulting cells suspensions were centrifuged down at 300 g for 5 min, followed by washing three times with AdDF+++ (Advanced DMEM/F12 supplemented with 1x Glutamax, 10 mM HEPES and 1x penicillin‐streptomycin). The cells were embedded into Matrigel (Mogengel Biotechnology) and plated in 20µL‐volume droplets on a 24‐well culture plates and allowed to solidify at 37 °C for 1 h before addition of full growth medium (AdDF+++ supplemented with 10% Noggin conditioned medium (U‐Protein Express), 10% R‐Spondin1 conditioned medium (made in‐house), 50× B27 supplement (Life Technologies), 10 mM nicotinamide (Sigma‐Aldrich), 1.25 mM N‐acetyl‐L‐cysteine (Sigma‐Aldrich), 10 µm ROCK inhibitor (Y‐27632)(Abmole), 500 nM A83‐01 (Tocris), 1 µm forskolin (Bio‐Techne), 10 ng mL^−1^ FGF10 (PeproTech), 50 ng mL^−1^ EGF (PeproTech) and 1000X Primocin (InvivoGen)). All pipette tips used in the above process were pre‐wetted with Anti‐Adherence Rinsing Solution (BioGenous) before usage. All procedures were approved by the Research Ethical Committee of Qilu Hospital of Shandong University (Approval number: KLYY‐202309‐055). All procedures had passed the scientific review by the Research Department of Qilu Hospital, Shandong University (Approval number: QLCR20230484).

### Lactobacillus Crispatus Strain 5578

Specialized collection swabs were collected from Qilu Hospital of Shandong University and immediately plated onto an MRS plate (Solarbio, Cat# M8330) and incubated at 37 °C in anaerobic conditions for 3–5 days. Bacterial growth from plates was sub‐cultured until single colonies could be isolated. 16S rRNA sequencing was used to identify the bacterial isolate species. *Lactobacillus crispatus* isolates were cultured in MRS broth at 37 °C in anaerobic conditions (10% CO2, 5% _H2_, nitrogen balance).

### PDO Co‐Cultures and Cell Co‐Cultures

- PDO & live *L. crispatus* co‐cultures: PDO and *L. crispatus* (1 × 10^9^) were vigorously shaken (37 °C, 5%CO2) in a solution with a total volume of 200 µL in an EP tube. The quantity of *L. crispatus* was calculated by measuring the OD600 value.
- PDO & dead *L. crispatus* co‐cultures: After exposing the *L. crispatus* to UV light for 2 h in a sterile workstation, 1× Penicillin‐Streptomycin was added and incubated overnight. PDO and dead *L. crispatus* (the same volume as live *L. crispatus*) were vigorously shaken (37 °C, 5%CO2) in a solution with a total volume of 200 µL in an EP tube.
- PDO & CFS/MRS co‐cultures: PDO and CFS/MRS (10%) were vigorously shaken (37 °C, 5%CO2) in a solution with a total volume of 200 µL in an EP tube (Figure 3A). PDO were cultured in Matrigel (37 °C, 5%CO2), and CFS/MRS were added to the culture medium outside the Matrigel with the concentration of 10% (Figure 3B–G).
- Cells co‐cultures: CFS/MRS were added to the culture medium (37 °C, 5%CO2) of cells with the concentration of 10%.

All procedures were approved by the Research Ethical Committee of Qilu Hospital of Shandong University (Approval number: KLYY‐202309‐055). All procedures had passed the scientific review by the Research Department of Qilu Hospital, Shandong University (Approval number: QLCR20230484).

### PH Test

The pH values of vaginal secretions were measured by clinical vaginal microecology testing. And the pH values of reagents in Table S3, Supporting Information were obtained by SevenExcellence Multiparameter. Before testing, the instrument must be calibrated with standard products. In addition, before testing each sample, the detection probe must be cleaned with sterile triple‐distilled water and dried. Each sample should be kept away from air as much as possible before testing to prevent changes in pH value. For example, if the RPMI 1640 medium (normal pH value is about 7.5) is exposed to air for too long, the pH value will increase.

### scRNA Sequencing

ScRNA sequencing, consisting of single‐cell dissociation, complementary DNA (cDNA) amplification, and library construction, was performed on the 10× Genomics Chromium Platform by LC‐Bio Technology co. ltd., (Hangzhou, China). First, after making sure the cell concentration to the ideal range, typically 700–1200 cells µL^−1^ using Countstar Rigel S2 (Countstar) to count, we confirmed that the samples passed the quality check. The gel beads containing barcode information were then mixed with the cell and enzyme mixture, and subsequently encapsulated by oil surfactant droplets in the microfluidic system by loading onto the Chromium Controller to form Gel Bead‐in‐Emulsions (GEMs). The GEMs were flowed into a collection reservoir where they were gathered, and the gel beads dissolved to release the barcode sequences. Second, 10× Barcoded cDNA was generated from single‐cell GEMs via reverse‐ transcription PCR and purified using Dyna beads MyOne SILANE magnetic beads (PN: 2 000 048, 10× Genomics). The gel beads were disrupted, and the oil droplets were broken to use cDNA as a template for PCR amplification. The products from all GEMs were pooled together to construct a standardized sequencing library. The cDNA was then fragmented by enzymatic digestion into ≈200–300 bp fragments, followed by the addition of sequencing adapters and primers as part of the conventional next‐generation sequencing library construction process. Finally, PCR amplification was performed to obtain the DNA library. High‐throughput sequencing was conducted using the dual‐end sequencing mode of the Illumina NovaSeq 6000 platform. The raw data were processed using the official 10× Genomics analysis software Cell Ranger (version 7.2.0) for data filtering, genome alignment, transcript quantification, and cell identification, ultimately resulting in a gene expression matrix for each cell. Subsequently, Seurat (version 4.1.1) was used for further cell filtering, normalization of cell expression quantification, classification of cell subpopulations, differential gene expression analysis for each subgroup, and marker gene selection. Additionally, Single R (version 4.1.2) was utilized for preliminary identification of cell types and single‐cell data mining of the clustered cells obtained from our sequencing.

### 16S rRNA Sequencing

16S rRNA gene sequencing methods were adapted from the methods developed for the Earth Microbiome Project and Human Microbiome Project performed by Novogene (Beijing, China) for library generation, sequencing, and data analyses. Bacterial genomic DNA was extracted using QIAamp Fast DNA Stool Mini Kit (QIAGEN). Briefly, the 16S rDNA V4 region was amplified by PCR using a 515F‐806R primer pair and sequenced on the MiSeq platform (Illumina) using the 2×250 bp paired‐end protocol. The primers used for amplification contain adapters for MiSeq sequencing and single‐end barcodes allowing pooling and direct sequencing of PCR products.

### Untargeted Metabolomics

This Metabolomics project performed by BMKGENE (Beijing, China) consisted of metabolite extraction, instrumental analysis, and qualitative and quantitative analysis of metabolites of 10 samples, basing on the LC‐QTOF platform. First, metabolite extraction included adding an appropriate volume of extraction solution and magnetic beads for grinding and sonication. Vacuum drying was needed after centrifugation and collection of the supernatant. An appropriate amount of extraction solution was added for reconstitution before analysis on the instrument. Second, the analysis platform of this project was the Waters Acquity I‐Class PLUS ultra‐high‐performance liquid chromatography coupled with the Waters Xevo G2‐XS QTOF high‐resolution mass spectrometer. Last, the raw data collected using MassLynx V4.2 was processed using Progenesis QI software for peak extraction, peak alignment, and other data processing operations. The Progenesis QI software with the online METLIN database, public databases, and a self‐built database by BMKGENE was used for Identification. Theoretical fragment identification was also conducted, with a mass deviation within 100 ppm for parent ions and within 50 ppm for fragment ions.

### Live/Dead Bacteria Dual Staining

Prior to staining, N01 dye and PI dye were each diluted 500‐fold with 0.85% NaCl solution at 37 °C to prepare the staining working solution. After washing the bacteria samples three times with 0.85% NaCl solution, mixed and resuspended it with 100—200 ul of staining working solution, and incubated it in the dark at room temperature for 15 min. Then, washed the samples once with 0.85% NaCl solution and observed and photographed them under a confocal scanning imaging microscope (Sunny CSIM 110).

### PDO Viral Transfection

When the organoids’ diameters reached 200 µm or more, mixed the lentivirus solution(MOI = 20) and Polybrene solution(1 µg mL^−1^) to non‐antibiotic medium. After incubating the organoids under the conditions of 37 °C, 5%CO_2_ with the above medium for 24 h, replaced it with fresh medium. Observed and photographed the organoids under a confocal scanning imaging microscope (Sunny CSIM 110) and fluorescence imaging microscope (Nikon ECLIPSE Ti).

### Cell Proliferation and Clonogenic Assays

Strictly followed the instructions of the Cell Counting Kit‐8 to measure the cell proliferation. Cultivated the cells in 96‐well plates, with ≈(2–5) × 103 cells per well. After treating the above‐mentioned cells with various conditions required for the experiment, replaced the original medium with new one containing 10% CCK8 stock solution and incubated them for 2–4 h. Finally, detected the absorbance at a wavelength of 450 nm. Counted the treated cells under experimental conditions required, and inoculated them into a six‐well plate at a density of 500 cells per well. Then cultivated them at 37 °C, 5%CO2 for 14days. After treating with methanol, the resulting clones were stained with crystal violet for 15 min. Each clone was considered standard when it contained more than 50 cells.

### Cell Migration and Invasion

The transwell assay was used to evaluate the migration and invasion capacity of cells. For migration, 7×104 treated cells were resuspended in 200 µL of FBS‐free medium. Then the cell suspension was added to the upper chambers. Subsequently, 750 µL of medium containing 10%FBS was added to the bottom chamber. The chambers were then incubated at 37 °C, 5% CO2 for about 8 h. The cells on the lower surface of the upper chamber were treated with methanol for 15 min. After that, the cells were stained with crystal violet solution for 15 min. For invasion, most of the process were the same as in the migration assay, except that before inoculating 14×104 cells, a mixture of 40–50 µL of Matrigel and medium was added on the membrane of the upper chamber, and the incubation time was increased to 16 h. The cells were observed and photographed using a light microscope (Nikon ECLIPSE Ti).

### Immunohistofluorescence

After dewaxing, rehydrating, and performing antigen retrieval, the tissue sections were treated with 0.2%Triton‐X 100 solution for permeabilization and 5%goat serum for antigen blocking. The primary antibodies were then added to the sections for overnight incubation at 4 °C. Subsequently, the corresponding fluorescent secondary antibodies were incubated at 37 °C for 1 h. Finally, the sections were stained with DAPI and phalloidin. Observed and photographed the images under a confocal scanning imaging microscope (Sunny CSIM 110) and fluorescence imaging microscope (Nikon ECLIPSE Ti).

### Live/Dead PDO Dual Staining

Scraped the organoids by pipette tips along with the matrigel from the bottom of the culture dish. After centrifugation at 300 ×g for 5 min, organoids were resuspended with PBS. After mixing the organoids with AO/PI Mix Dye, incubated the solution at 37 °C for 5 min. Counted and photographed the organoids under the Fluorescence Cell Counter (MONWEI SmartCell 800).

### WB

At 4 °C, the cells were lysed with RIPA lysis buffer containing 1% PMSF for 30 min. The samples were then centrifuged to obtain the supernatant. And the protein concentration was assessed with the Enhanced BCA Protein Assay Kit. The protein samples were separated using the PAGE Gel Fast Preparation Kit (PG112, Epizyme Biotech), with a total of 30 µg of protein per well. Subsequently, the proteins were transferred to polyvinylidene fluoride (PVDF) membranes (Merck, ISEQ00010) using the BIO‐RAD Trans‐blot apparatus at 350 mA for 120 min. The PVDF membranes, which had been blocked with Blot Blocking Buffer, were then incubated with primary antibodies at 4 °C overnight. Afterward, the membranes were incubated with secondary antibodies at room temperature for 1 h. The band signal was detected by the Immobilon Western Chemiluminescent HRP Substrate (Merck) in conjunction with the Image Quant LAS4000 system (General Electric Company). The endogenous controlled protein in this study was β‐Actin.

### Lipid Peroxidation

The C11‐BODIPY 581/591 Lipid Peroxidation Sensor was employed to ascertain cellular lipid peroxidation levels, thereby quantifying ferroptosis. Cells were seeded at a density of 2 × 10^4^ cells per well in 20mm‐glass‐bottom culture dishes (Biofil). Diluted the stock solution of C11 BODIPY 581/591 to a concentration of 10 µm with medium. Incubated the cells which had been added the solution above at 37 °C, 5%CO2 for 30 min. Observed and photographed the cells under a confocal scanning imaging microscope (Sunny CSIM 110).

### Reactive Oxygen Species

The Reactive Oxygen Species Assay Kit was used to measure the level of intracellular ROS. Cells were seeded at a density of 2 × 10^4^ cells per well in 20mm‐glass‐bottom culture dishes (Biofil). After diluting DCFH‐DA 1000‐fold with serum‐free medium, added the solution of 1 ml to each culture dish. After being stained by Hoechst 33 342 for nucleus, observed and photographed the cells which had been incubated at 37 °C, 5%CO2 for 20 min under a confocal scanning imaging microscope (Sunny CSIM 110).

### Organoid Viability ATP Assay

Organoid Viability ATP Assay Kit was employed to ascertain the proliferation vitality of organoids. After digesting, live/dead staining, and counting, the organoids were passaged and plated of the same density in 96‐well plates. After the organoids grew to a certain diameter, various treatments of this study were applied to them. Mixed the medium and detection reagent in a 1:1 ratio and added the solution to the Matrigel containing the organoids. Measured the luminescence of the samples which had been incubated at room temperature for 20 min after vigorous shaking of 1000 rpm, 5 min.

### 5‐Ethynyl‐2′‐Deoxyuridine

Cells were seeded at a density of 1 × 10^3^ cells per well in 96‐well plates. After being treated, the cells were fixed with 4% paraformaldehyde and then permeabilized with 0.3%Triton X‐100. The Cell‐Light EdU Apollo567 In Vitro Kit was employed to ascertain the DNA replication vigor of cells. After treating the cells strictly following the instructions manual, observed and photographed the cells under a fluorescence imaging microscope (Nikon ECLIPSE Ti).

### Lipid Peroxidation MDA Assay

The MDA levels in the cells were measured using the Lipid Peroxidation MDA Assay Kit. After digestion and processing, the cells were centrifuged at 12 000 g for 10 min, and the supernatant was collected. A portion of the supernatant was used to determine the protein concentration, while the remaining portion was mixed with the detection reagent according to the kit instructions and heated at 100 °C for 15 min. After cooling to room temperature, the mixture was centrifuged at 1000 g for 10 min. Subsequently, 200 µL of the supernatant were transferred to a microplate reader, and the absorbance was measured at 532 nm. The MDA concentration was calculated by substituting the absorbance value into the standard curve, and the final MDA concentration per unit protein mass was obtained by dividing this value by the protein concentration.

### 4‐HNE ELISA Assay

The cellular 4‐HNE level was determined using a competitive ELISA assay. Microplates were coated with 4‐HNE antigen. 4‐HNE in the samples competed with the coated 4‐HNE for binding sites on biotin‐labeled anti‐4‐HNE monoclonal antibodies. Unbound components were washed away. Horseradish peroxidase (HRP)‐labeled avidin was then added, allowing specific binding between biotin and avidin to form immune complexes, followed by another wash to remove unbound substances. The chromogenic substrate TMB was added, producing a blue color under HRP catalysis, which turned yellow after adding a stop solution. The optical density (OD) at 450 nm was measured using a microplate reader. The 4‐HNE concentration inversely correlated with the OD450 value, and sample 4‐HNE concentrations were calculated by the standard curve.

### A‐CoA ELISA Assay

The A‐CoA levels in cells were determined using a double‐antibody sandwich ELISA. Anti‐A‐CoA antibodies were coated onto the microplate, allowing A‐CoA in the samples to bind to the coated antibodies during the experiment. Subsequently, biotinylated anti‐A‐CoA antibodies and horseradish peroxidase (HRP)‐labeled avidin were sequentially added. The anti‐A‐CoA antibodies bound to the A‐CoA already attached to the coated antibodies, while biotin specifically interacted with avidin to form immune complexes. Unbound components were washed away. Upon addition of the chromogenic substrate TMB, a blue color developed under the catalytic action of HRP, which changed to yellow after adding the stop solution. The optical density (OD) at 450 nm was measured using a microplate reader. The A‐CoA concentration was proportional to the OD450 value, and the sample concentration was calculated using a standard curve.

### Fatty Acid Oxidation (NADH) Colorimetric Assay

The level of FAO is reflected by detecting NADH levels. During the FAO process, substrates and NAD+ are consumed to generate NADH. NADH reacts with electron coupling agents and chromogenic agents to produce an orange‐red substance, which is measured at 450 nm. The NADH level can be determined by analyzing the sample's OD value. The NADH concentration is calculated by substituting the OD value into a standard curve, and dividing this value by the protein concentration of the sample yields the NADH level per unit protein mass.

### GSH and GSSG Assay

This experiment used glutathione reductase to reduce GSSG into GSH, while GSH reacted with the chromogenic substrate DTNB to produce yellow TNB and GSSG. By properly preparing the reaction system, combining the two sequential reactions allowed total glutathione (GSSG + GSH) to act as the rate‐limiting factor for color development. The amount of total glutathione determined the quantity of yellow TNB formed, enabling the calculation of total glutathione levels by measuring absorbance at 412 nm (A412). Using appropriate reagents to first eliminate GSH in the sample, the same reaction principle can then be applied to quantify GSSG content. By subtracting the measured GSSG content from the total glutathione (GSSG + GSH), the GSH concentration can be determined.

### Statistical Analysis

Statistical analyses were conducted using GraphPad Prism 10.4.2. Data were presented as means ± standard deviation (SD). Statistical comparisons between two groups were performed using paired Student it‐test. Statistical comparisons between more than two groups were performed with One‐way analysis of variance (ANOVA). Statistical comparisons between two curves were compared by Two‐way analysis of variance test. *p* values < 0.05 were considered to be statistically significant. Statistical details of experiments can be found in the legends.

## Conflict of Interest

The authors declare no conflict of interest.

## Author Contributions

Y.Z. Lead contact Conceptualization: Q.Z., S.L., and Y.Z.; software: Q.Z. and D.Y.; formal analysis: Q.Z.; investigation: Q.Z., Y.X., Y.X., Y.Z., X.L., S.L., and Y.Z.; resources: S.L. and Y.Z.; data curation: Q.Z.; writing – original draft: Q.Z. and S.L; writing – review and editing: Q.Z., S.L., Y.X., Y.X., X.L., Y.Z., D.Y., S.H., and Y.Z.; visualization: Q.Z.; supervision: S.L. and Y.Z.; project administration: S.L. and Y.Z.; funding acquisition: S.L. and Y.Z.

## Supporting information



Supporting Information

## Data Availability

The data that support the findings of this study are available from the corresponding author upon reasonable request.
