# Supplementary material for: Erucic Acid, Derived by Lactobacillus Crispatus, Induces Ferroptosis in Cervical Cancer Organoids Through the PPAR‐δ Signaling Pathway
Source: Adv Sci (Weinh). 2025 Oct 13;12(48):e12599. doi: 10.1002/advs.202512599 (PMC12752652; doi:10.1002/advs.202512599)

**Supplementary Table 1.** Patient Characteristics (N=112).

^a)^ Interquartile range. ^b)^ Body mass index. ^C)^ The International Federation of Gynecology and Obstetrics. ^d)^ Adenocarcinoma and adenosquamous carcinoma.

|  | **Cervical Cancer Patients (*n*=23)** | | **Healthy Cervix Patients**  **(*n*=89)** | |
| --- | --- | --- | --- | --- |
|  | ***N* (%)** | **Median (IQR^a^)** | ***N* (%)** | **Median (IQR)** |
| **Age (years)** |  | 45 (38-54) |  | 35 (26-44) |
| **BMI^b^ (kg/m^2^)** |  | 23.7 (21.9-26.6) |  | 24.6 (21.9-28.3) |
| **Smoking Status** |  |  |  |  |
| **Never** | 20 (87.0) |  | 87 (97.8) |  |
| **Former** | 2 (8.7) |  | 2 (2.2) |  |
| **Current** | 1 (4.3) |  | 0 |  |
| **FIGO 2009 Stage^c^** |  |  |  |  |
| **I** | 20 (87.0) |  |  |  |
| **II** | 3 (13.0) |  |  |  |
| **III-IV** | 0 |  |  |  |
| **Histology** |  |  |  |  |
| **Non-squamous^d^** | 0 |  |  |  |
| **Squamous** | 23 (100.0) |  |  |  |
| **Other Disease** |  |  |  |  |
| **Endometrial Polyp** |  |  |  | 38 (42.7) |
| **Leiomyoma of Uterus** |  |  |  | 27 (30.3) |
| **Ovarian Cyst** |  |  |  | 23 (25.8) |
| **Other** |  |  |  | 1 (1.1) |

**Supplementary Table 2.** Inclusion Criteria and Exclusion Criteria of different patients.

| **Biological Samples** | **Patient Type** | **Inclusion Criteria** | **Exclusion Criteria** |
| --- | --- | --- | --- |
| **Vaginal Secretions** | Cervical cancer | Pathological diagnosis of cervical cancer. | 1.Colpitis  2.Antibiotic usage within the preceding three months |
|  | Healthy Cervix | Both TCT and HPV tests were negative. | 1.Colpitis  2.Antibiotic usage within the preceding three months |
| **Cervical Cancer Tissue** | Cervical cancer | Pathological diagnosis of cervical cancer. | 1.Tumor dimension is smaller than 1cm*1cm*1cm  2.Have received radiotherapy, chemotherapy, immunotherapy or other treatment due to cervical cancer |

**Supplementary** **Table 3.** The pH value of reagents.

| **Reagents** | **PH value** |
| --- | --- |
| Complete RPMI 1640 medium | 7.509 |
| 100% MRS | 6.321 |
| 100% CFS | 4.448 |
| 10% MRS (diluted by complete RPMI 1640 medium) | 7.764 |
| 10% CFS (diluted by complete RPMI 1640 medium) | 7.631 |
| 9.872% CFS (diluted by complete RPMI 1640 medium) | 7.764 |

**Supplementary Figure 1.** Identification of *L. crispatus (LC 001).*

(**A**) BLAST Analysis of Sequences Derived from Forward Primer Sequencing: *Lactobacillus crispatus* strain 8090 16S ribosomal RNA gene, partial sequence. Sequence ID: [MT464262.1](https://www.ncbi.nlm.nih.gov/nucleotide/MT464262.1?report=genbank&log$=nuclalign&blast_rank=1&RID=KW1JF8EV016). Length:1475. Number of Matches: 1. Range 1: 7 to 1005.

(**B**) BLAST Analysis of Sequences Derived from Reverse Primer Sequencing: *Lactobacillus crispatus* strain 5578 16S ribosomal RNA gene, partial sequence. Sequence ID: [MT510321.1](https://www.ncbi.nlm.nih.gov/nucleotide/MT510321.1?report=genbank&log$=nuclalign&blast_rank=1&RID=KW1Y17DG016). Length:1470. Number of Matches: 1. Range 1: 472 to 1464.

| **A Alignment statistics for match #1** | | | | |
| --- | --- | --- | --- | --- |
| Score | Expect | Identities | Gaps | Strand |
| 1666 bits(902) | 0.0 | 969/1001(97%) | 5/1001(0%) | Plus/Plus |
|  |  |  |  |  |

Query 2 GGGGGGCG-GTCTATAC-TGCAGTCGAGCGAGCGGAACTAACAGATTTACTTCGGCAATG 59

|||||||| | |||||| ||||||||||||||||||||||||||||||||||||| ||||

Sbjct 7 GGGGGGCGTGCCTATACATGCAGTCGAGCGAGCGGAACTAACAGATTTACTTCGGTAATG 66

Query 60 ACGTTAGGAAAGCGAGCGGCGGATGGGTGAG-AACGCGTGGGGAACCTGCCCCATAGTCT 118

||||||||||||||||||||||||||||||| ||| ||||||||||||||||||||||||

Sbjct 67 ACGTTAGGAAAGCGAGCGGCGGATGGGTGAGTAACACGTGGGGAACCTGCCCCATAGTCT 126

Query 119 GGGATACCACTTGGAAACAGGTGCTAATACCGGATAAGAAAGCAGATCGCATGATCACCT 178

||||||||||||||||||||||||||||||||||||||||||||||||||||||||| ||

Sbjct 127 GGGATACCACTTGGAAACAGGTGCTAATACCGGATAAGAAAGCAGATCGCATGATCAGCT 186

Query 179 TTTAAAAGGCGGCGTACGTTGTCCCTATGGGATGGCCGCGCGGTGCATTAGCGAGTTGGT 238

|||||||||||||||| | |||| ||||||||||||| |||||||||||||| |||||||

Sbjct 187 TTTAAAAGGCGGCGTAAGCTGTCGCTATGGGATGGCCCCGCGGTGCATTAGCTAGTTGGT 246

Query 239 AGGGGAAAGGCTTACCAAGGCGATGATGCGTATTCCATTTGTTAGACTGGCCGGCCGCGT 298

| || |||||||||||||||||||||||| || | | ||| |||||| ||||| | |

Sbjct 247 AAGGTAAAGGCTTACCAAGGCGATGATGCATAGCCGAGTTGAGAGACTGATCGGCCACAT 306

Query 299 TGGGACTGAGACACGGTCCAACTTTATACGGGAGGCAGCAGTAGGGAATCTTCCACAATG 358

|||||||||||||||| |||| | ||||||||||||||||||||||||||||||||||

Sbjct 307 TGGGACTGAGACACGGCCCAAACTCCTACGGGAGGCAGCAGTAGGGAATCTTCCACAATG 366

Query 359 GACGCAAGTCTGATGGAGCAACGCCGCGTGAGTGAAGAAGGTTTTCGGATCGTAAAGCTC 418

||||||||||||||||||||||||||||||||||||||||||||||||||||||||||||

Sbjct 367 GACGCAAGTCTGATGGAGCAACGCCGCGTGAGTGAAGAAGGTTTTCGGATCGTAAAGCTC 426

Query 419 TGTTGTTGGTGAAGAAGGATAGAGGTAGTAACTGGCCTTTATTTGACGGTAATCAACCAG 478

||||||||||||||||||||||||||||||||||||||||||||||||||||||||||||

Sbjct 427 TGTTGTTGGTGAAGAAGGATAGAGGTAGTAACTGGCCTTTATTTGACGGTAATCAACCAG 486

Query 479 AAAGTCACGGCTAACTACGTGCCAGCAGCCGCGGTAATACGTAGGTGGCAAGCGTTGTCC 538

||||||||||||||||||||||||||||||||||||||||||||||||||||||||||||

Sbjct 487 AAAGTCACGGCTAACTACGTGCCAGCAGCCGCGGTAATACGTAGGTGGCAAGCGTTGTCC 546

Query 539 GGATTTATTGGGCGTAAAGCGAGCGCAGGCGGAAGAATAAGTCTGATGTGAAAGCCCTCG 598

||||||||||||||||||||||||||||||||||||||||||||||||||||||||||||

Sbjct 547 GGATTTATTGGGCGTAAAGCGAGCGCAGGCGGAAGAATAAGTCTGATGTGAAAGCCCTCG 606

Query 599 GCTTAACCGAGGAACTGCATCGGAAACTGTTTTTCTTGAGTGCAGAAGAGGAGAGTGGAA 658

||||||||||||||||||||||||||||||||||||||||||||||||||||||||||||

Sbjct 607 GCTTAACCGAGGAACTGCATCGGAAACTGTTTTTCTTGAGTGCAGAAGAGGAGAGTGGAA 666

Query 659 CTCCATGTGTAGCGGTGGAATGCGTAGATATATGGAAGAACACCAGTGGCGAAGGCGGCT 718

||||||||||||||||||||||||||||||||||||||||||||||||||||||||||||

Sbjct 667 CTCCATGTGTAGCGGTGGAATGCGTAGATATATGGAAGAACACCAGTGGCGAAGGCGGCT 726

Query 719 CTCTGGTCTGCAACTGACGCTGAGGCTCGAAAGCATGGGTAGCGAACAGGATTAGATACC 778

||||||||||||||||||||||||||||||||||||||||||||||||||||||||||||

Sbjct 727 CTCTGGTCTGCAACTGACGCTGAGGCTCGAAAGCATGGGTAGCGAACAGGATTAGATACC 786

Query 779 CTGGTAGTCCATGCCGTAAACGATGAGTGCTAAGTGTTGGGAGGTTTCCGCCTCTCAGTG 838

||||||||||||||||||||||||||||||||||||||||||||||||||||||||||||

Sbjct 787 CTGGTAGTCCATGCCGTAAACGATGAGTGCTAAGTGTTGGGAGGTTTCCGCCTCTCAGTG 846

Query 839 CTGCAGCTAACGCATTAAGCACTCCGCCTGGGGAGTACGACCGCAAGGTTGAAACTCAAA 898

||||||||||||||||||||||||||||||||||||||||||||||||||||||||||||

Sbjct 847 CTGCAGCTAACGCATTAAGCACTCCGCCTGGGGAGTACGACCGCAAGGTTGAAACTCAAA 906

Query 899 GGAATTGACGGGGGCCCGCACAAGCGGTGGAGCATGTGGGTTTAATTCGAAGCAACGCGA 958

||||||||||||||||||||||||||||||||||||| ||||||||||||||||||||||

Sbjct 907 GGAATTGACGGGGGCCCGCACAAGCGGTGGAGCATGT-GGTTTAATTCGAAGCAACGCGA 965

Query 959 AGAACCCTTACCAGGTCTTGACATCTAGTGCCATTTGTAGA 999

|||| ||||||||||||||||||||||||||||||||||||

Sbjct 966 AGAA-CCTTACCAGGTCTTGACATCTAGTGCCATTTGTAGA 1005

| **B Alignment statistics for match #1** | | | | |
| --- | --- | --- | --- | --- |
| Score | Expect | Identities | Gaps | Strand |
| 1825 bits(988) | 0.0 | 993/995(99%) | 2/995(0%) | Plus/Minus |

Query 6 TGACTGCTTAGACGGCTCCTTCCCGAAGGTTAGGCCACCGGCTTTGGGCATTGCAGACTC 65

||||||||||||||||||||||||||||||||||||||||||||||||||||||||||||

Sbjct 1464 TGACTGCTTAGACGGCTCCTTCCCGAAGGTTAGGCCACCGGCTTTGGGCATTGCAGACTC 1405

Query 66 CCATGGTGTGACGGGCGGTGTGTACAAGGCCCGGGAACGTATTCACCGCGGCGTGCTGAT 125

||||||||||||||||||||||||||||||||||||||||||||||||||||||||||||

Sbjct 1404 CCATGGTGTGACGGGCGGTGTGTACAAGGCCCGGGAACGTATTCACCGCGGCGTGCTGAT 1345

Query 126 CCGCGATTACTAGCGATTCCAGCTTCGTGCAGTCGAGTTGCAGACTGCAGTCCGAACTGA 185

||||||||||||||||||||||||||||||||||||||||||||||||||||||||||||

Sbjct 1344 CCGCGATTACTAGCGATTCCAGCTTCGTGCAGTCGAGTTGCAGACTGCAGTCCGAACTGA 1285

Query 186 GAACAGCTTTCAGAGATTCGCTTGCCTTCGCAGGCTCGCTTCTCGTTGTACTGCCCATTG 245

||||||||||||||||||||||||||||||||||||||||||||||||||||||||||||

Sbjct 1284 GAACAGCTTTCAGAGATTCGCTTGCCTTCGCAGGCTCGCTTCTCGTTGTACTGCCCATTG 1225

Query 246 TAGCACGTGTGTAGCCCAGGTCATAAGGGGCATGATGACTTGACGTCATCCCCACCTTCC 305

||||||||||||||||||||||||||||||||||||||||||||||||||||||||||||

Sbjct 1224 TAGCACGTGTGTAGCCCAGGTCATAAGGGGCATGATGACTTGACGTCATCCCCACCTTCC 1165

Query 306 TCCGGTTTGTCACCGGCAGTCTCATTAGAGTGCCCAACTTAATGCTGGCAACTAATAACA 365

||||||||||||||||||||||||||||||||||||||||||||||||||||||||||||

Sbjct 1164 TCCGGTTTGTCACCGGCAGTCTCATTAGAGTGCCCAACTTAATGCTGGCAACTAATAACA 1105

Query 366 AGGGTTGCGCTCGTTGCGGGACTTAACCCAACATCTCACGACACGAGCTGACGACAGCCA 425

||||||||||||||||||||||||||||||||||||||||||||||||||||||||||||

Sbjct 1104 AGGGTTGCGCTCGTTGCGGGACTTAACCCAACATCTCACGACACGAGCTGACGACAGCCA 1045

Query 426 TGCACCACCTGTCTTAGCGTCCCCGAAGGGAACTTTGTATCTCTACAAATGGCACTAGAT 485

||||||||||||||||||||||||||||||||||||||||||||||||||||||||||||

Sbjct 1044 TGCACCACCTGTCTTAGCGTCCCCGAAGGGAACTTTGTATCTCTACAAATGGCACTAGAT 985

Query 486 GTCAAGACCTGGTAAGGTTCTTCGCGTTGCTTCGAATTAAACCACATGCTCCACCGCTTG 545

||||||||||||||||||||||||||||||||||||||||||||||||||||||||||||

Sbjct 984 GTCAAGACCTGGTAAGGTTCTTCGCGTTGCTTCGAATTAAACCACATGCTCCACCGCTTG 925

Query 546 TGCGGGCCCCCGTCAATTCCTTTGAGTTTCAACCTTGCGGTCGTACTCCCCAGGCGGAGT 605

||||||||||||||||||||||||||||||||||||||||||||||||||||||||||||

Sbjct 924 TGCGGGCCCCCGTCAATTCCTTTGAGTTTCAACCTTGCGGTCGTACTCCCCAGGCGGAGT 865

Query 606 GCTTAATGCGTTAGCTGCAGCACTGAGAGGCGGAAACCTCCCAACACTTAGCACTCATCG 665

||||||||||||||||||||||||||||||||||||||||||||||||||||||||||||

Sbjct 864 GCTTAATGCGTTAGCTGCAGCACTGAGAGGCGGAAACCTCCCAACACTTAGCACTCATCG 805

Query 666 TTTACGGCATGGACTACCAGGGTATCTAATCCTGTTCGCTACCCATGCTTTCGAGCCTCA 725

||||||||||||||||||||||||||||||||||||||||||||||||||||||||||||

Sbjct 804 TTTACGGCATGGACTACCAGGGTATCTAATCCTGTTCGCTACCCATGCTTTCGAGCCTCA 745

Query 726 GCGTCAGTTGCAGACCAGAGAGCCGCCTTCGCCACTGGTGTTCTTCCATATATCTACGCA 785

||||||||||||||||||||||||||||||||||||||||||||||||||||||||||||

Sbjct 744 GCGTCAGTTGCAGACCAGAGAGCCGCCTTCGCCACTGGTGTTCTTCCATATATCTACGCA 685

Query 786 TTCCACCGCTACACATGGAGTTCCACTCTCCTCTTCTGCACTCAAGAAAAACAGTTTCCG 845

||||||||||||||||||||||||||||||||||||||||||||||||||||||||||||

Sbjct 684 TTCCACCGCTACACATGGAGTTCCACTCTCCTCTTCTGCACTCAAGAAAAACAGTTTCCG 625

Query 846 ATGCAGTTCCTCGGTTAAGCCGAGGGCTTTCACATCAGACTTATTCTTCCGCCTGCGCTC 905

||||||||||||||||||||||||||||||||||||||||||||||||||||||||||||

Sbjct 624 ATGCAGTTCCTCGGTTAAGCCGAGGGCTTTCACATCAGACTTATTCTTCCGCCTGCGCTC 565

Query 906 GCTTTACGCCCAATAAATCCGGACAACGCTTGCCACCTACGTATTACCGCGGCTGCTGGC 965

||||||||||||||||||||||||||||||||||||||||||||||||||||||||||||

Sbjct 564 GCTTTACGCCCAATAAATCCGGACAACGCTTGCCACCTACGTATTACCGCGGCTGCTGGC 505

Query 966 ACGTAGTTAGCCCGTGACTTTCTGGGTTGATTACC 1000

|||||||||||| |||||||||||| |||||||||

Sbjct 504 ACGTAGTTAGCC-GTGACTTTCTGG-TTGATTACC 472

**Supplementary Figure 2.** The bright views on D0 and D21 of organoids which were treated with 5%MRS, 5%CFS, 10%MRS,10%CFS,15%MRS and 15%CFS. Scale bar, 500μm.


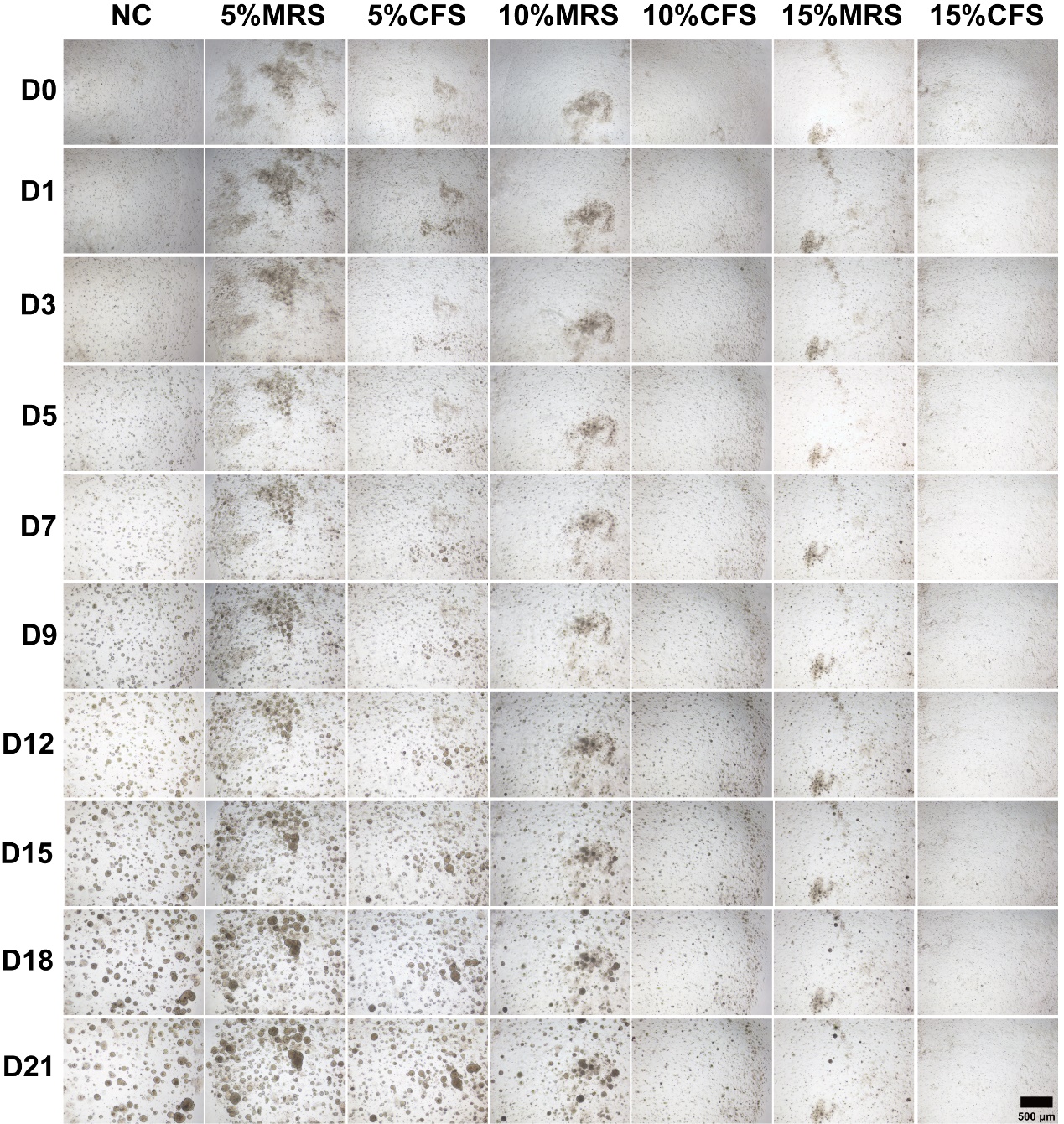


**Supplementary Figure 3. (A, C)** The pseudotime analysis of seurat clusters. **(B, D)** The pseudotime analysis of new cell clusters basing on cell types.


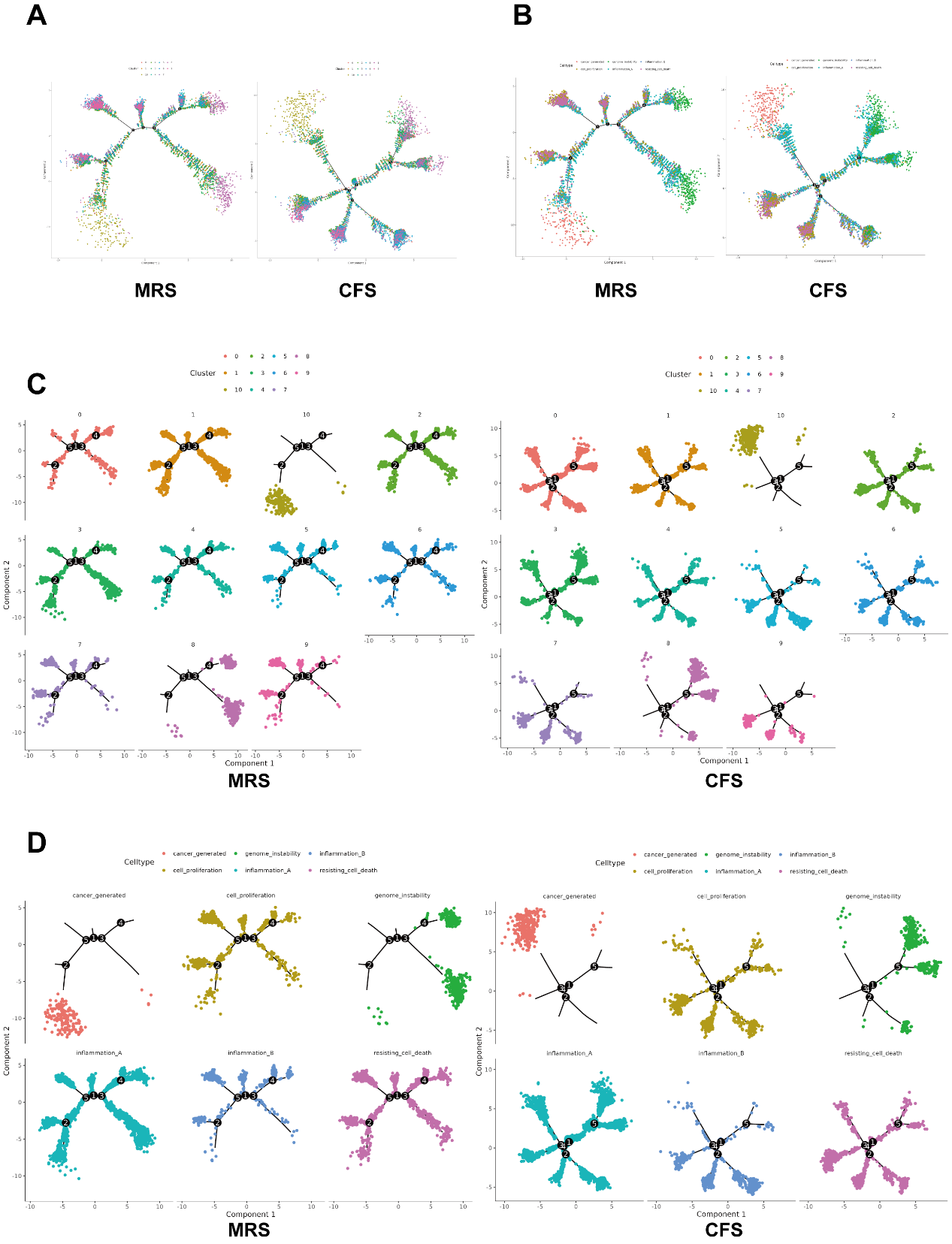


**Supplementary Figure 4. (A)** The cell morphology of Ca Ski, C-33A, Hela and H8 cervical cell lines co-cultured with 10% CFS. **(B)** The cell morphology of Ca Ski treated with erucic acid of different concentration gradients respectively. **(C)** The CCK-8 assays of Ca Ski and C-33A cells treated with erucic acid of different concentration gradients respectively.


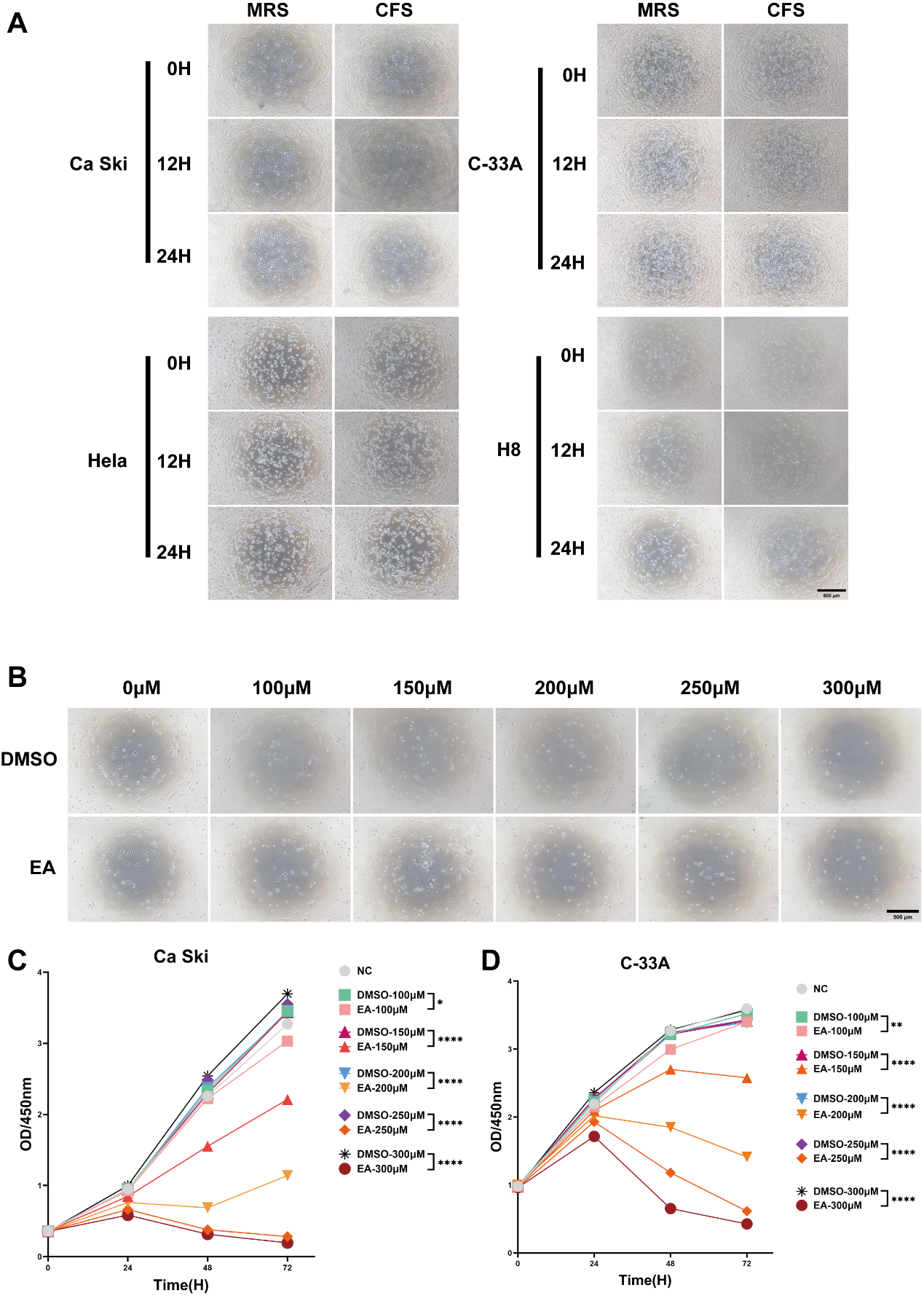


**Supplementary Figure 5.** **(A, B, C, D, E)** Bar chart of the Western Blot results (fold change) relating to FAO of Ca Ski cells treated with 10%PBS, 10%MRS, 10%CFS, 10%CFS+DMSO, 10%CFS+GSK3787, 10%CFS+Fer-1, Blank, DMSO, EA, EA+DMSO, EA+ GSK3787, EA+Fer-1. Statistical analyses: One-way analysis of variance, ns p>0.05, *p<0.05, **p<0.01, ***p<0.001, ****p<0.0001. **(F, G, H, I, J)** Bar chart of the Western Blot results (fold change) relating to ferroptosis of Ca Ski cells treated with 10%PBS, 10%MRS, 10%CFS, 10%CFS+DMSO, 10%CFS+GSK3787, 10%CFS+Fer-1, Blank, DMSO, EA, EA+DMSO, EA+ GSK3787, EA+Fer-1. Statistical analyses: One-way analysis of variance, ns p>0.05, *p<0.05, **p<0.01, ***p<0.001, ****p<0.0001. **(K, L, M, N, O, P, Q, R)** Bar chart of the Western Blot results (fold change) relating to proliferation and HPV of Ca Ski cells treated with 10%PBS, 10%MRS, 10%CFS, 10%CFS+DMSO, 10%CFS+GSK3787, 10%CFS+Fer-1, Blank, DMSO, EA, EA+DMSO, EA+ GSK3787, EA+Fer-1. Statistical analyses: One-way analysis of variance, ns p>0.05, *p<0.05, **p<0.01, ***p<0.001, ****p<0.0001.


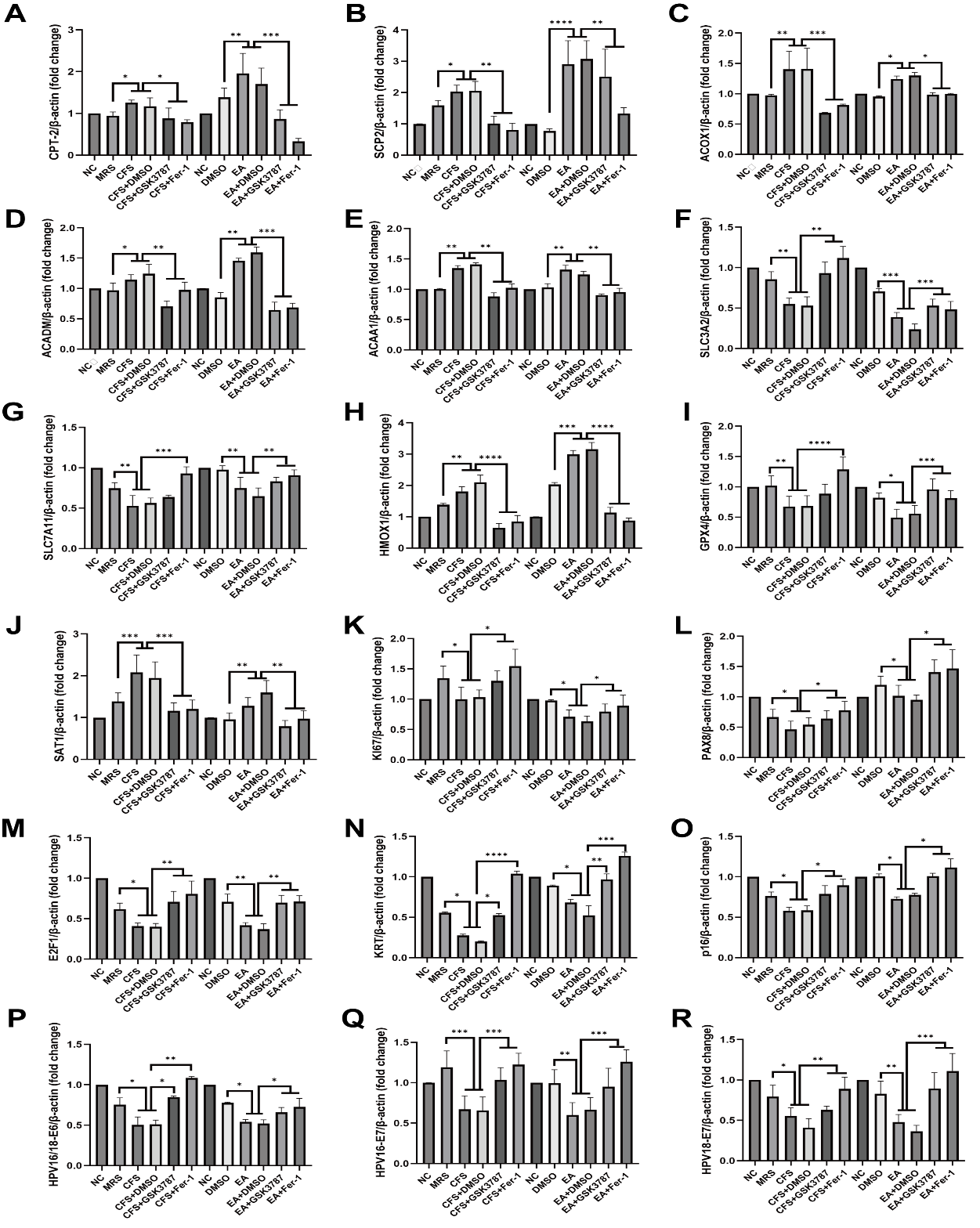

Supplement: Supplementary file 1 — Supporting Information [file ADVS-12-e12599-s001.docx]
